# Broad-Spectrum Extracellular Antiviral Properties of Cucurbit[*n*]urils

**DOI:** 10.1021/acsinfecdis.2c00186

**Published:** 2022-09-05

**Authors:** Luke M. Jones, Elana H. Super, Lauren J. Batt, Matteo Gasbarri, Francesco Coppola, Lorraine M. Bhebhe, Benjamin T. Cheesman, Andrew M. Howe, Petr Král, Roger Coulston, Samuel T. Jones

**Affiliations:** † Department of Materials and The Henry Royce Institute, The University of Manchester, Manchester M19 3PL, United Kingdom; ‡ Institute of Materials, Interfaculty Bioengineering Institute, MXG 030 Lausanne, Switzerland; § Department of Chemistry, University of Illinois at Chicago, Chicago, Illinois 60607, United States; ∥ Aqdot Limited, Iconix Park, London Road, Pampisford, Cambridge CB22 3EG, United Kingdom; ⊥ Department of Physics and Department of Biopharmaceutical Sciences, University of Illinois at Chicago, Chicago, Illinois 60607, United States

**Keywords:** cucurbituril, antiviral, virucidal, virustatic, macrocycle, dose−response

## Abstract

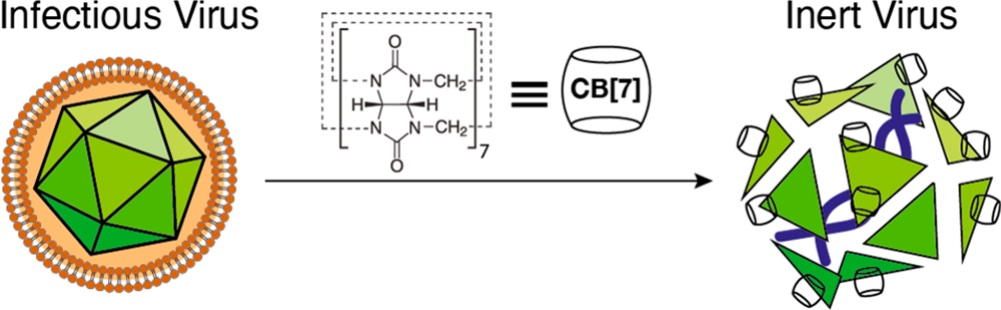

Viruses are microscopic pathogens capable of causing disease and are responsible for a range of human mortalities and morbidities worldwide. They can be rendered harmless or destroyed with a range of antiviral chemical compounds. Cucurbit[*n*]urils (CB[*n*]s) are a family of macrocycle chemical compounds existing as a range of homologues; due to their structure, they can bind to biological materials, acting as supramolecular “hosts” to “guests”, such as amino acids. Due to the increasing need for a nontoxic antiviral compound, we investigated whether cucurbit[*n*]urils could act in an antiviral manner. We have found that certain cucurbit[*n*]uril homologues do indeed have an antiviral effect against a range of viruses, including herpes simplex virus 2 (HSV-2), respiratory syncytial virus (RSV) and SARS-CoV-2. In particular, we demonstrate that CB[7] is the active homologue of CB[*n*], having an antiviral effect against enveloped and nonenveloped species. High levels of efficacy were observed with 5 min contact times across different viruses. We also demonstrate that CB[7] acts with an extracellular virucidal mode of action via host–guest supramolecular interactions between viral surface proteins and the CB[*n*] cavity, rather than via cell internalization or a virustatic mechanism. This finding demonstrates that CB[7] acts as a supramolecular virucidal antiviral (a mechanism distinct from other current extracellular antivirals), demonstrating the potential of supramolecular interactions for future antiviral disinfectants.

Viruses are harmful, microscopic pathogens that replicate within host cells before being spread via aerosols (coughs, sneezes), surface contamination, and/or infected biological fluids. The impact each has varies, but they can be responsible for severe disease in human hosts and disease outbreaks (epidemics or pandemics), including the COVID-19 pandemic. Epidemics of viral diseases are also increasing in frequency^1,2^ with associated costs in overall mortality, morbidity, and economic damage.^3,4^ Viruses themselves are composed of amino acids self-assembled into proteinaceous shells, capsids, protecting the genome. The capsid may or may not be surrounded by a lipid envelope. Regardless of envelope presence, all viruses sport attachment, or spike, proteins on their surface for the purpose of adhering to and infecting host cells.^5^

To address the global threat posed by all viruses, there are three main interventions: vaccines, drugs, or disinfection. Chemical disinfectants (such as bleach and soaps) are often broad-spectrum and have been deployed in a range of sprays, surface sanitizers, and hand gels to prevent viral spread.^6^ However, some of these disinfectants, such as bleach, can be harmful to human health, the environment, or textile quality. Less overtly damaging antiviral options have been previously studied, including quaternary ammonium compounds,^7,8^ nanoparticles,^9−11^ and polymers.^12−14^ Each works differently: some target the genetic material and some the viral capsid proteins, but all aim to inhibit the virus such that it is no longer infectious. However, many of these compounds retain a degree of toxicity^15,16^ (environmental^17^ or otherwise), limiting their effective use.

Macrocyclic compounds are frequently used as drug carriers,^18,19^ including as carriers for antivirals.^20,21^ Additionally, due to their wide range of binding motifs, macrocycles offer the potential to interact directly with viruses and inhibit infection. However, to date, the use of macrocycles as antivirals has been limited to modified macrocycles^22^ or via indirect effects (for example, via binding cholesterol^23^). Cucurbit[*n*]urils (CB[*n*]s) are barrel-shaped macrocycles composed of “*n*” glycoluril monomers/units linked by methylene bridges^24^ that are produced in a range of ring sizes, which can then be separated into discrete sizes from *n* = 5–8 to 10.^25−27^ Like other macrocycles, CB[*n*]s have a cavity capable of supramolecular binding to a range of guest molecules^28^ with the larger CBs (CB[8] and CB[10]) capable of including two guests simultaneously.^29,30^ This binding arises via guest interactions with the hydrophobic, unpolarizable cavity and the polar, carbonyl-rich portals and can be supported by an entropy gain on releasing water molecules from the cavity.^25^ CBs have been widely studied and utilized to produce unique supramolecular structures for a range of applications in drug delivery,^31,32^ tuning protein functionality,^33^ and forming hydrogels.^34,35^ CBs have also been shown to bind to an array of biologically relevant groups, including the amino acid constituents^36,37^ of proteins such as insulin.^38^ Recently, it has been suggested that cucurbit[7]uril (CB[7]) is able to act as an indirect antiviral by binding intracellularly to polyamines and disrupting the viral replication cycle.^39^

We hypothesized that CB[*n*]s could bind directly to viruses via supramolecular interactions between exposed surface proteins and the CB cavity, which would then inhibit infection. This inhibition could be via reversible binding (virustatic) or may be irreversible if binding results in an irreversible conformational change (virucidal). Both possibilities would inhibit infection, but only a virucidal mechanism would lead to inactivation of the virus. This interaction is different from many other extracellular antivirals, which largely function by mimicking cell-binding regions^22,40,41^ or leaching toxic metal ions.^42,43^ In order to test our hypotheses, we used a range of viruses, culturable *in vitro*, and performed a number of different assays to determine the effectiveness and mode of action of the individual CB homologues, CB[6], CB[7], and CB[8], in addition to the *n* = 6, 7, and 8 CB[*n*] substance.

## Results

### Investigating Antiviral Efficacy

**Figure 1:**
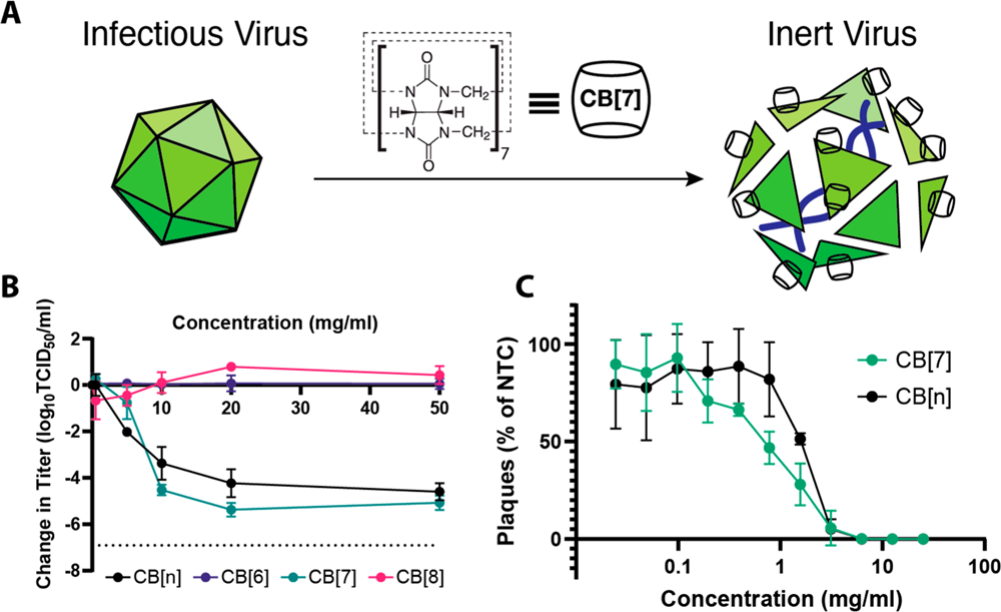
(A) Graphic illustration depicting the virucidal antiviral effect of CB[7] with a generic virus (not to scale). (B) Data from TCID_50_ assays with various CBs against HSV-2, showing a decrease in HSV-2 titer when mixed with CB[*n*] and CB[7] but not with CB[6] and CB[8] (*n* = 3). (C) Dose–response assay against HSV-2 (*n* = 3), indicating that the IC_50_ value for CB[7] is lower than that for CB[*n*]. In all instances, dotted lines indicate limits of detection and error bars indicate standard deviations.

We began with solution-based testing to determine if any of our CB homologues exhibited antiviral efficacy. To investigate the efficacy of CB[*n*]s as antivirals, we needed to identify an easily culturable model virus/cell system. For this, we selected herpes simplex virus-2 (HSV-2) in Vero cells, which is a well-studied system across a range of assays, is easily cultured *in vitro*, and readily forms plaques in cell monolayers. In order to determine if CB[*n*]s were able to inhibit viral infections, median tissue culture infectious dose (TCID_50_) assays were used with HSV-2. These assays were performed in Dulbecco’s Modified Eagle Medium (DMEM) media, a complex media containing amino acids, vitamins, and salts that are capable of binding to the CB portal and/or cavity. CB homologue substances CB[6], CB[7], and CB[8], and CB[*n*], were studied. Each CB sample was mixed with virus for 5 min before being serially diluted to determine viral titer. The initial TCID_50_ assays indicated that both CB[*n*] and CB[7] were effective at inhibiting HSV-2, with an approximately 5-log reduction in viral titer observed at 20–50 mg/mL. The lack of viral titer reduction observed in conjunction with CB[6] and CB[8] indicated that it was the CB[7] component of the CB[*n*] responsible for the reduction (Figure 1B). CB[*n*] and CB[7] were both significantly different from CB[6] and CB[8] (all adjusted *p* values < 0.0001). CB[7] is more soluble in aqueous systems than CB[6] and CB[8],^44,45^ meaning more will be available to bind to the virus. In addition, the cavity of CB[6] may be too small for binding to amino acids^46^ with the same ease as CB[7] (though it can form supramolecular peptide interactions^47^). Conversely, the cavity of CB[8] is certainly large enough to bind to and has an affinity for particular amino acids.^48^ More experiments would be needed to determine why CB[8] lacks antiviral efficacy. The effectiveness of CB[*n*] being lower than that of CB[7] is likely due to the fact that CB[*n*] contains a majority of the less effective CB[6] and CB[8] homologues.

Once the antiviral nature of CB[*n*] and CB[7] had been established, we used dose–response assays to quantify the effect. Such assays allow the half maximal inhibitory concentration (IC_50_) to be determined. Dose–response assays show that CB[*n*] has an IC_50_ of 1.5 mg/mL and CB[7] has an IC_50_ of 1.3 mg/mL (Figure 1C). Some cellular damage was observed at the highest concentrations of CB[7] and CB[*n*], though this dissipated rapidly as the dilution increased. CB[6] and CB[8] were not tested in this experiment as they had previously not shown any antiviral effect and were therefore considered unlikely to give a useful IC_50_ value.

**Figure 2:**
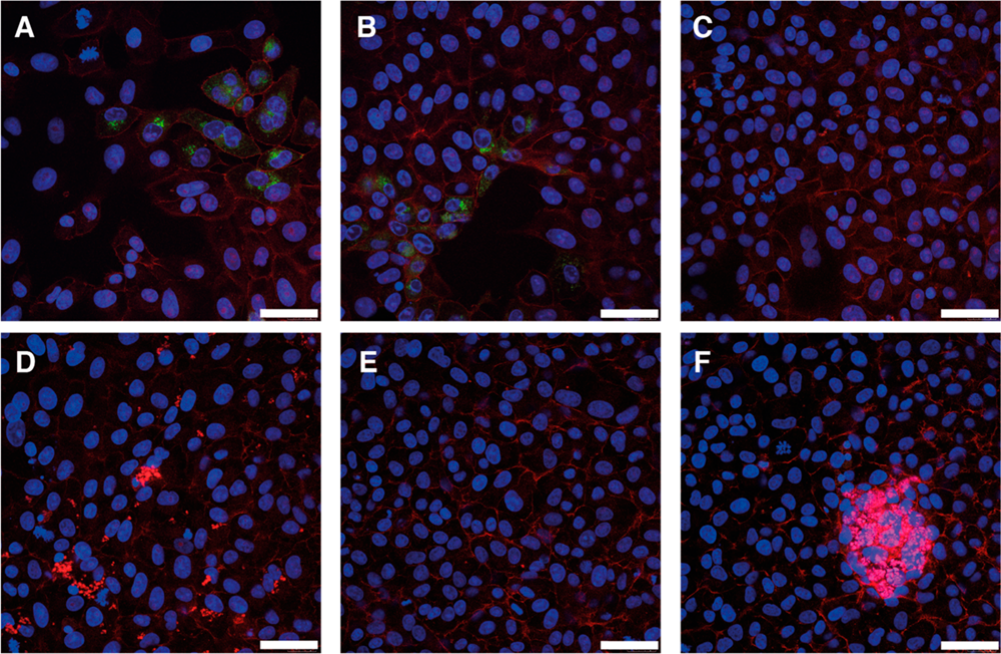
HSV-2 was mixed with different concentrations of CB[7] (A: 0 mg/mL; B: 0.5 mg/mL; C: 5 mg/mL; D: 10 mg/mL; E: 20 mg/mL; F: 50 mg/mL) and applied to cells before incubating for 24 h. HSV-2 in green, phalloidin in red, and cell nuclei in blue. Scale bar: 50 μm.

Additional experiments were performed via confocal fluorescence to confirm the effectiveness of CB[7] and CB[*n*] against HSV-2. This study showed that, while virus was still detectable following a 1:1 solution mixture of 0 and 0.5 mg/mL CB[7], at higher levels (5 mg/mL and above), no virus was detected following incubation with the cells (Figure 2). In addition, when virus was mixed with CB[*n*], no virus was detected at 10 mg/mL and above, although increasing cellular damage, perhaps due to undissolved solid particles, is observed at 20 mg/mL and particularly 50 mg/mL (see Figure S1). At higher concentrations of CB[7] and CB[*n*], large red clusters can be observed atop the cell monolayer (Figure 2F); we believe these to be undissolved sedimentations of CB that exhibit autofluorescence, as they are not present at lower concentrations. Although no viral presence was observed via confocal microscopy at 5 mg/mL CB[7], TCID_50_ assays indicate some will still be present. This discrepancy can be explained by the fact that 5 mg/mL CB[7] is associated with an approximately 0.8-log reduction, which may be enough to reduce the virus to a level in which none was observed within the five blind-selected field-of-views of the monolayer.

### Mode of Action

**Figure 3:**
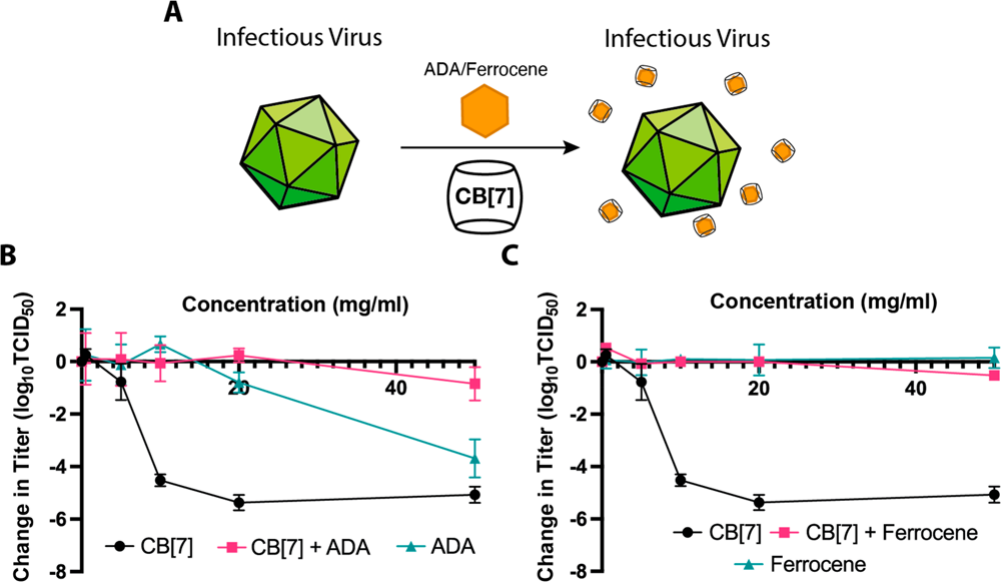
(A) Graphic illustration depicting the ability of ADA or ferrocene to block the cavity of CB[7] and eliminate the antiviral effect. (B) Data from the TCID_50_ assays, illustrating the drop in titer when HSV-2 is exposed to CB[7] (*n* = 3) or ADA alone (*n* = 2), but no decrease in titer is observed when HSV-2 is mixed with CB[7] + ADA (*n* = 2). (C) Data from the TCID_50_ assays indicates that CB[7] + ferrocene (*n* = 1) and ferrocene alone (*n* = 3) have no antiviral effect.

In order to confirm that inhibition is due to supramolecular binding of the CB to the virus, we performed an additional assay, mixing CB[7] with 1-adamantylamine (ADA). ADA has one of the strongest binding affinities (1.7 ± 0.8 × 10^14^ M^–1^)^49^ with CB[7], meaning the CB[7] cavity would be occupied and no binding to the virus could occur (Figure 3A). Different concentrations of 1:1 CB[7]/ADA were prepared and mixed with virus followed by TCID_50_ assays to determine viral titer. We observed that the 1:1 mix of CB[7]/ADA had no antiviral efficacy over the entire range (Figure 3B). Interestingly, ADA has an antiviral efficacy of its own;^50,51^ this seems to be based on blocking a key viral protein in influenza viruses.^52^ However, when mixed at an equimolar ratio with CB[7], the antiviral efficacy of both compounds is neutralized due to the encapsulation of the ADA by CB[7], and therefore, it is unavailable to bind to and inhibit the virus. We performed a similar experiment with ferrocene, another strong binder with the CB[7] cavity (>10^6^ M^–1^)^53,54^ and one that lacks any antiviral properties. Complexation with ferrocene similarly eliminated the antiviral efficacy of CB[7] (Figure 3C), reinforcing our hypothesis that the cavity of CB[7] is fundamental to its antiviral properties.

In order to understand better the interactions between cucurbit[*n*]uril molecules and HSV-2, we performed molecular dynamics (MD) simulations of CB[6], CB[7], and CB[8] coupled with glycoprotein B (gB) located on the HSV-2 envelope capsid.^55^ Glycoprotein B together with other glycoproteins present on the envelope of HSV have already been studied and found to be responsible for both viral attachment (gB, gC, and gD) and fusion with the cell plasma membrane (gB, gD, gH, and gL).^56^ We decided to select gB as a possible target due to its interesting dual role as well as other works showing that blocking this glycoprotein decreases viral infectivity.^57^ Each simulation was performed by randomly placing 6 copies of each CB near the gB protein, which had a fixed backbone and free residues (Figure S2). The systems were simulated (see Methods) with NAMD2^58^ and CHARMM36^59^ force fields in a physiological solution (0.15 M NaCl).

**Figure 4:**
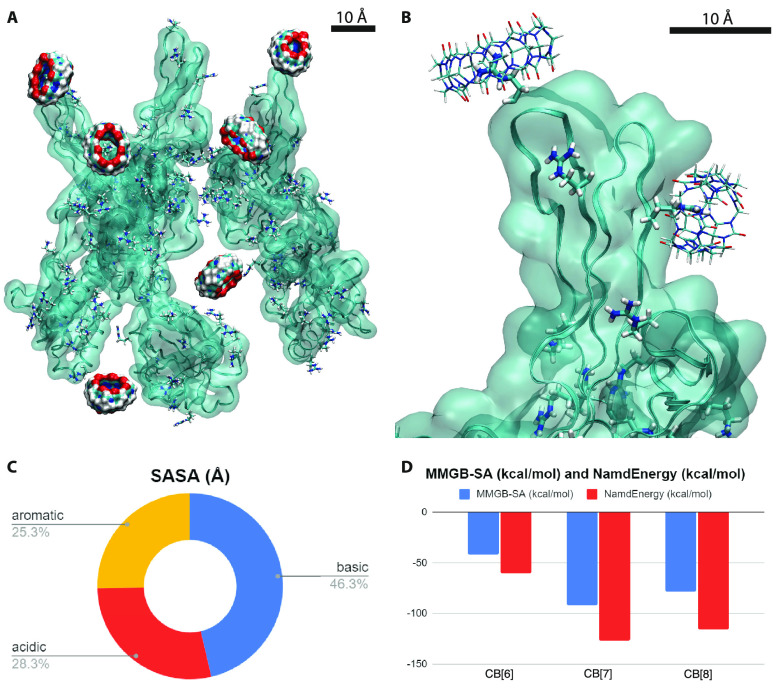
(A) Simulation snapshot where 4 out of 6 CB[7] rings interact with the LYS and ARG in HSV-2. (B) CB[7] encapsulating an ARG at the top and a LYS at the bottom. (C) Solvent accessible surface area (SASA) calculated for different amino acids exposed on the surface of the HSV-2 gB. (D) Averages of MMGB-SA and Namd energy calculations on the last 20 ns.

The CB rings provide multiple possibilities for binding to different amino acid residues. One possibility is a dipole–dipole interaction between the carbonyl groups and positively charged residues. Alternatively, the hollow rings with large openings can encapsulate less polar amino acids and interact with them through van der Waals coupling (Figure 4A,B). In a physiological solution, CB[7] is known to preferentially bind with aromatic amino acids by the second mechanism.^60^ However, our simulations (100 ns) for CB[7] revealed that it mostly binds with the basic amino acids arginine and lysine (ARG, LYS), which are present on the gB surface.

To better understand this behavior, we calculated the solvent accessible surface area (SASA) of basic, aromatic, and acidic residues exposed on the gB surface. Figure 4C shows that the basic amino acids are more accessible than the more hydrophobic aromatic residues, which are mostly buried in gB or poorly exposed. We also calculated the overall SASA for the amino acids ARG and LYS against that of the aromatic amino acids, and after the result is normalized, the basic side chains have a value of 129.1 Å^2^ while the aromatic ones have a value of 47.7 Å^2^. Therefore, although CB[7] and CB[8] may prefer to bind to aromatic amino acids, the more accessible basic residues can significantly contribute to the interaction between gB and CBs.

Next, we wanted to understand better why CB[7] acts antivirally in previous assays but CB[6] and CB[8] do not. We have calculated the average coupling energies between the different CB homologues and the side chains of gB proteins. The energies were averaged over the 6 rings (normalized to one ring) during the last 20 ns from the 100 ns of our simulations. Figure 4D shows the direct (van der Waals and Coulombic) coupling energies (with the dielectric constant of water of 80.4) and the MMGB-SA^61^ coupling free energies (see Methods). Both results show that CB[7] binds the strongest and most often to gB, while CB[6] binds half as much as CB[7] and CB[8] binds slightly better than CB[6] due to its larger structure. We can see that 4 out of 6 CB[7] rings remain anchored to the basic amino acids, while 2/6 remain coupled for CB[6] and 1/6 for CB[8] rings (Figures S3 and S4 display the CB[6] and CB[8] rings on gB). Though these results demonstrate the importance of CB[7] for inhibiting the HSV-2 virus, the exact mechanism of action for this inhibition is unknown. Thus, here, we hypothesize two possible mechanisms for the disruption of viral infection: first, blocking the electrostatic interaction between the positively charged residues on the viral gB with the negatively charged cellular heparan sulfate (HS) groups (viral attachment to cell step); alternatively, CB[7] binding may prevent the interaction of gB with one of its receptors, paired immunoglobulin-like receptor (PILRα), nonmuscle myosin heavy chain (NMHC-IIA), or myelin-associated glycoprotein (MAG)^56^ by encapsulating the basic side chains of gB.

**Figure 5:**
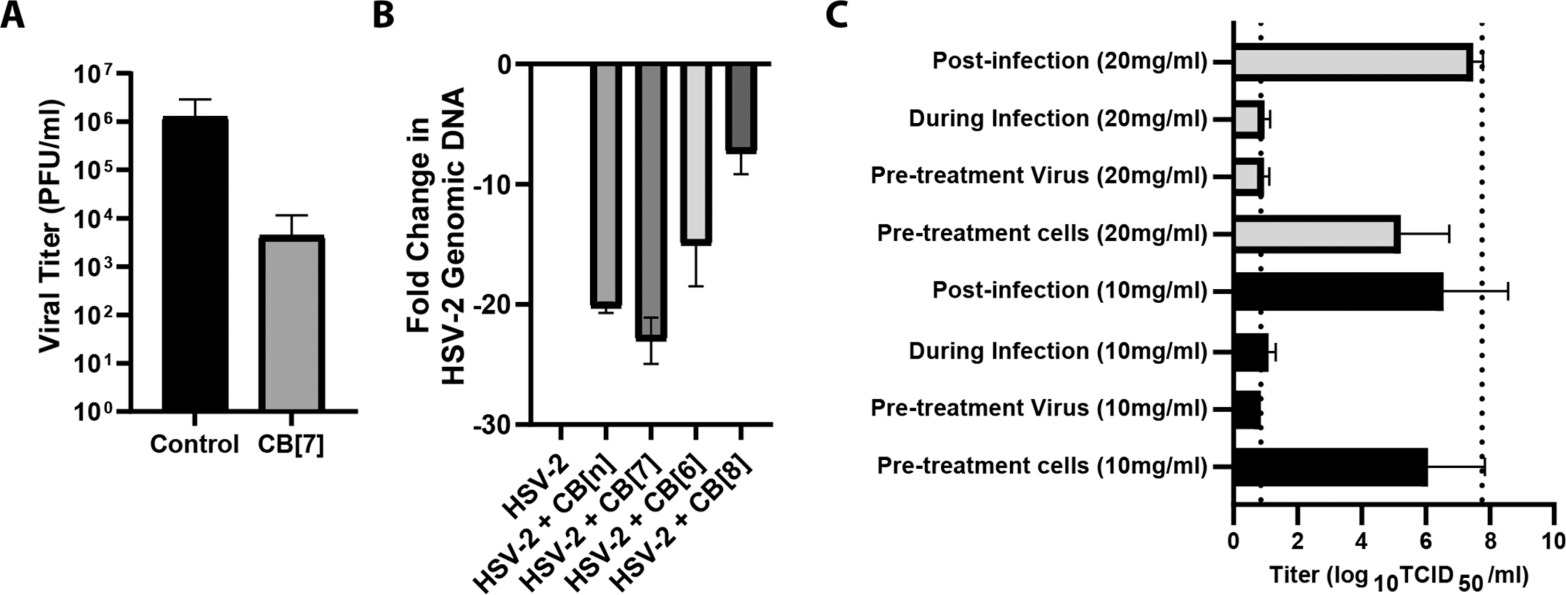
(A) Virucidal assay data showing a 2-log reduction in viral titer with CB[7], indicating CB[7] acts virucidally (*n* = 3). (B) DNA-exposure assay shows that CB[7] and CB[*n*] are associated with the release of DNA from the virus. (C) Time of addition study suggests that CB[7] only inhibits HSV-2 titer when added simultaneously with virus to the cells and not when added before or after viral exposure (10 mg/mL in black, 20 mg/mL in gray) (*n* = 3).

While previous assays confirm that CB[7] has antiviral properties and can bind to viral proteins (MD simulations), the exact mode of action was still unclear. CB[7] has been previously suggested to work by cellular internalization and binding to polyamines.^39^ In order to further elucidate the antiviral mechanism, virucidal assays were performed with CB[7] against HSV-2. A virucidal assay is used to distinguish between destructive (virucidal) and nondestructive (virustatic) interactions between antivirals and viruses. In this instance, CB[7] was mixed with HSV-2 for 60 min before being serially diluted across a 96-well plate. If the interactions between the CBs and virus were reversible nondestructive binding events, then serial dilution would cause removal of the CB from the viral surface and viral plaques would be observed at low dilutions. However, we observe that after serial dilution there is no recovery of virus and a greater than 2-log reduction in viral titer, indicating that the binding of CB[7] to HSV-2 is indeed virucidal (Figure 5A). We also observe a virucidal mechanism with other viruses (*vide infra*).

In order to confirm that the mode of action of the CB was extracellular, DNA exposure assays were performed. Such assays confirm the breakdown of the HSV-2 viral capsid and release of the genome in a cell-free environment. This experiment is critical to show that the mechanism is not via disruption of intracellular processes, such as viral replication pathways. The genome exposure assay includes a DNase treatment step after CB exposure; if the genome has been released (as would be the case for a virucide), it will be accessible to further enzymatic degradation and thus unable to be amplified and detected via qPCR. Conversely, any viruses left intact by antiviral treatment will retain their genome, which can be detected via qPCR amplification. Data from this experiment shows that CB[*n*] and CB[7] were associated with significant drops in genome detection (*p* values of <0.0001 for both), suggesting that CB[7] does degrade the virion capsid and expose the genome to DNase treatment. The CB[8] and CB[6] treatment were also both significantly different from the virus with no treatment control (*p* values of 0.02 and 0.0005, respectively) (Figure 5B) with CB[6] treatment in particular being associated with an unexpected drop in genomic DNA detection. We initially hypothesized that CB[6] and CB[8] traces were interfering with the qPCR and making it appear as though there was a drop in genome detection. However, subsequent controls suggested that, although a high enough CB concentration can affect the qPCR results, not enough CB was present in any sample tested to change the Ct value (see Figure S5A,B). This behavior was investigated by adding various concentrations of CB[6], CB[7], and CB[8] to qPCR master mixes and observing the impact on the Ct value (we showed higher concentrations of all CBs are associated with higher Ct values). In addition, a positive control was performed using *Pseudomonas* DNA in qPCR master mixes and adding the same volume of extracted viral DNA (that had initially been exposed to CB treatment). This combination had no effect on the Ct value. It is important to note that this assay illustrates that cucurbiturils can interact virucidally with viruses in a completely cell-free environment.

To elucidate further the antiviral mode of action of CBs, a series of time of addition studies was performed. We observed an antiviral effect when CB[7] was added directly to cells simultaneously with virus or when virus stock was pretreated with CB[7]. However, we observed no viral inhibition when the CB[7] was added to the cells and later removed (cell monolayers washed twice with PBS) prior to viral introduction. In addition, the assay we performed allowed for infection followed by a period of CB exposure (to allow for potential CB–cell internalization) before removal of the CB prior to incubation and viral replication/plaque formation. No antiviral effect was observed, further indicating that the CB binds directly to the virus in suspension (Figure 5C) with a virucidal mechanism. In the 10 mg/mL treatment, the titer for cells treated postinfection was significantly different from that when the virus was pretreated with CB[7] or when the CB[7] and virus were added simultaneously (*p* values of 0.0072 and 0.003, respectively). The equivalent *p* values for the 20 mg/mL treatments were 0.0002 and <0.0001. These assays strongly suggest that, in these systems at least, CB[7] does not disrupt the viral replication cycle following cellular internalization, as previously reported.^39^

### Broad Spectrum Efficacy

**Figure 6:**
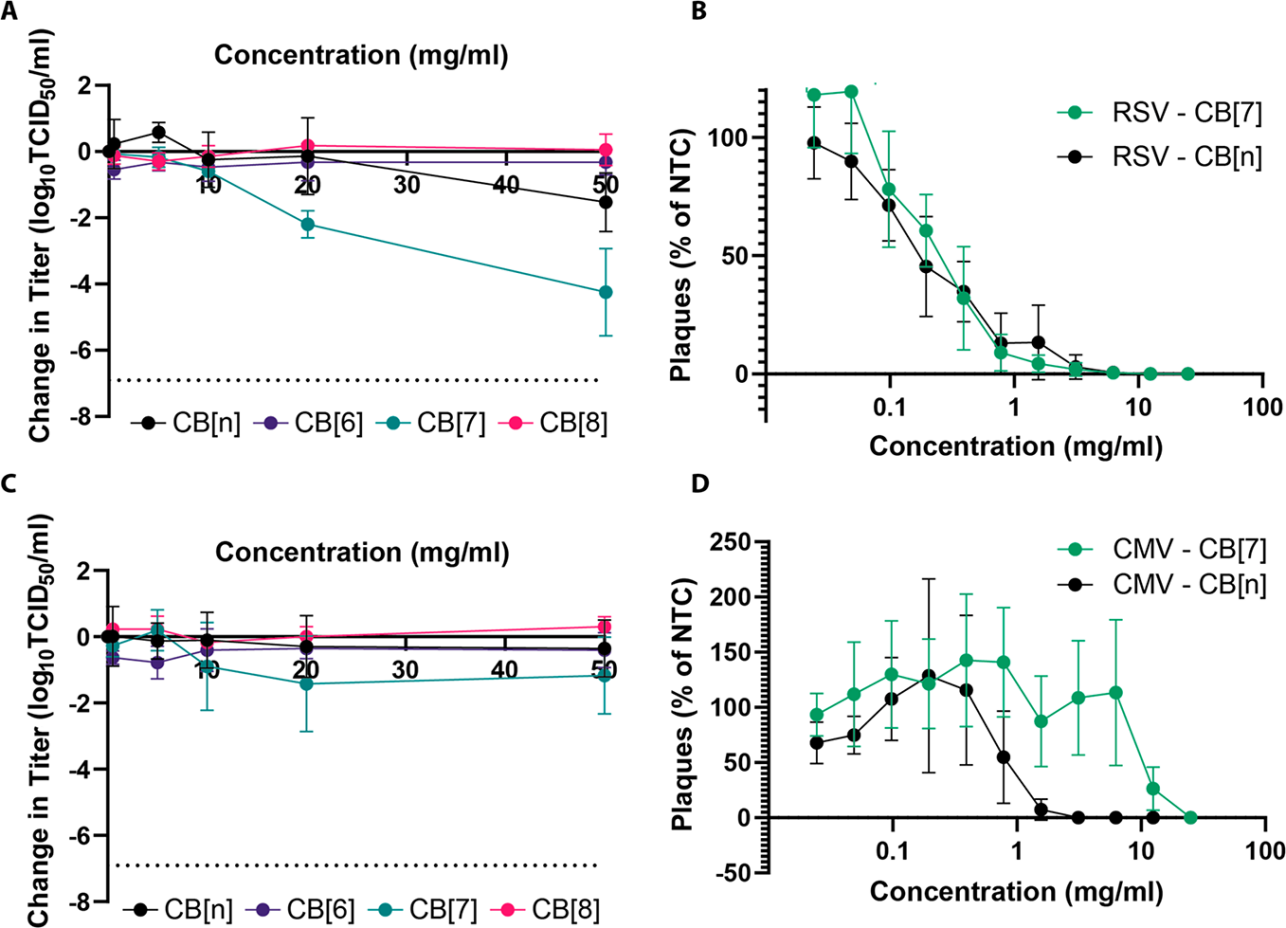
(A) TCID_50_ assays against RSV showed that CB[7] and CB[*n*] are associated with large reductions in titer, particularly above 20 mg/mL (*n* = 3). (B) Dose–response assays reconfirmed the effectivity of both CB[7] and CB[*n*] with both being seemingly similarly effective when utilized in this assay (*n* = 3). (C) A limited reduction in titer was observed via the TCID_50_ assay when CMV was mixed with different concentrations of CB[7]. CB[6], CB[8], and CB[*n*] appeared ineffective (*n* = 3). (D) Dose–response experiments with CMV showed that CB[7] and CB[*n*] display limited antiviral properties when tested in this manner (*n* = 4 for both).

Having confirmed that CBs are effective antivirals against HSV-2, we also investigated if they were antiviral against other viruses. We began by testing CB[*n*] and CB[7] against respiratory syncytial virus (RSV). TCID_50_ assays (with a 5 min contact time between virus and antiviral, as previously performed) confirmed that both were also antiviral against RSV with a 2–3-log reduction in viral titer at the highest concentrations. As previously observed, CB[6] and CB[8] were ineffective (Figure 6A), and the difference on average between CB[7] and CB[6]/CB[8] was statistically significant (adjusted *p* value < 0.0001 for both). This was again quantified more precisely via dose–response assays, which showed that CB[7] has an IC_50_ value of 0.16 mg/mL and CB[*n*] also has an IC_50_ value of 0.16 mg/mL (Figure 6B). Virucidal assays were also performed with RSV and showed a 2-log reduction in viral titer (Figure S6), indicating that CB[7] has a virucidal mode of action for both RSV and HSV-2. Cucurbiturils were also tested against cytomegalovirus (CMV) and seemed to have an antiviral effect when used in TCID_50_ assays: as seen previously, CB[7] showed the most efficacy (Figure 6C). CB[7] titer reductions were significantly different from those with CB[8] (adjusted *p* value of 0.0161), but otherwise, there were no significant differences detected between cucurbiturils. In addition, both CB[7] and CB[*n*] seemed to be relatively ineffective when used in a dose–response assay with IC_50_ values of 10.40 and 0.78 mg/mL, respectively (Figure 6D).

Murine norovirus strain 1 (MNV1) (a surrogate for pathogenic human norovirus) was also investigated. Unlike previous viruses tested, MNV1 is a nonenveloped virus. TCID_50_ assays demonstrated that no cucurbituril (including CB[7] and CB[*n*]) functions as an effective antiviral against this virus (Figure S7). Subsequent statistical analysis found no significant difference between any cucurbituril homologue used in this experiment. Dose–response assays could not be performed for murine norovirus as it did not form plaques in the same manner as other viruses tested. Further experiments would be needed to determine why CB[*n*] proved ineffective against this virus, especially as efficacy was observed against a different MNV strain (MNV Berlin S99) (*vide infra*).

**Figure 7:**
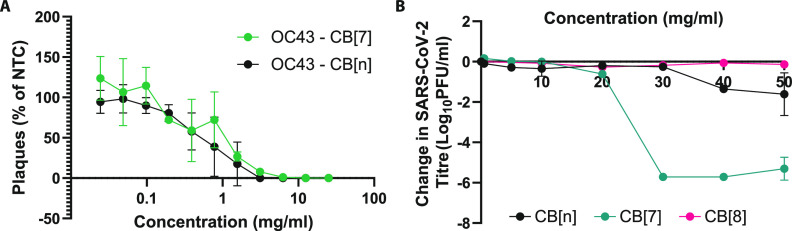
(A) Dose–response assay performed against human coronavirus OC43. CB[7] and CB[*n*] appear similarly effective (*n* = 3 for both). (B) CB[7] greatly reduces the SARS-CoV-2 viral titer at 30 mg/mL concentration and above (*n* = 2). CB[*n*] displays a more limited antiviral effect over the same concentration range (*n* = 2). CB[8] has no effect (*n* = 1).

Human coronaviruses are viruses of potential future pandemic concern. Therefore, we investigated the effects of CB[*n*] against two human coronaviruses: human coronavirus (strain OC43) and SARS-CoV-2 (Switzerland/GE9586/2020). OC43 was tested against CB[7] and CB[*n*] in a dose–response assay. Both CB[7] and CB[*n*] showed considerable efficacy against OC43 with IC_50_ values of 0.65 and 2.8 mg/mL, respectively (Figure 7A). The efficacy of cucurbiturils against SARS-CoV-2, the human coronavirus responsible for the COVID-19 pandemic, was also investigated. Titration assays against SARS-CoV-2 demonstrated that CB[*n*] and CB[7] are both antiviral against SARS-CoV-2 but to different extents. CB[*n*] has a more limited efficacy, even at a 50 mg/mL concentration, while CB[7] is highly effective (approximately 6-log reduction) at 30 mg/mL and above. CB[8] has no antiviral effect (Figure 7B). These experiments suggest that CB[7] has broad efficacy against different human coronaviruses.

**Figure 8:**
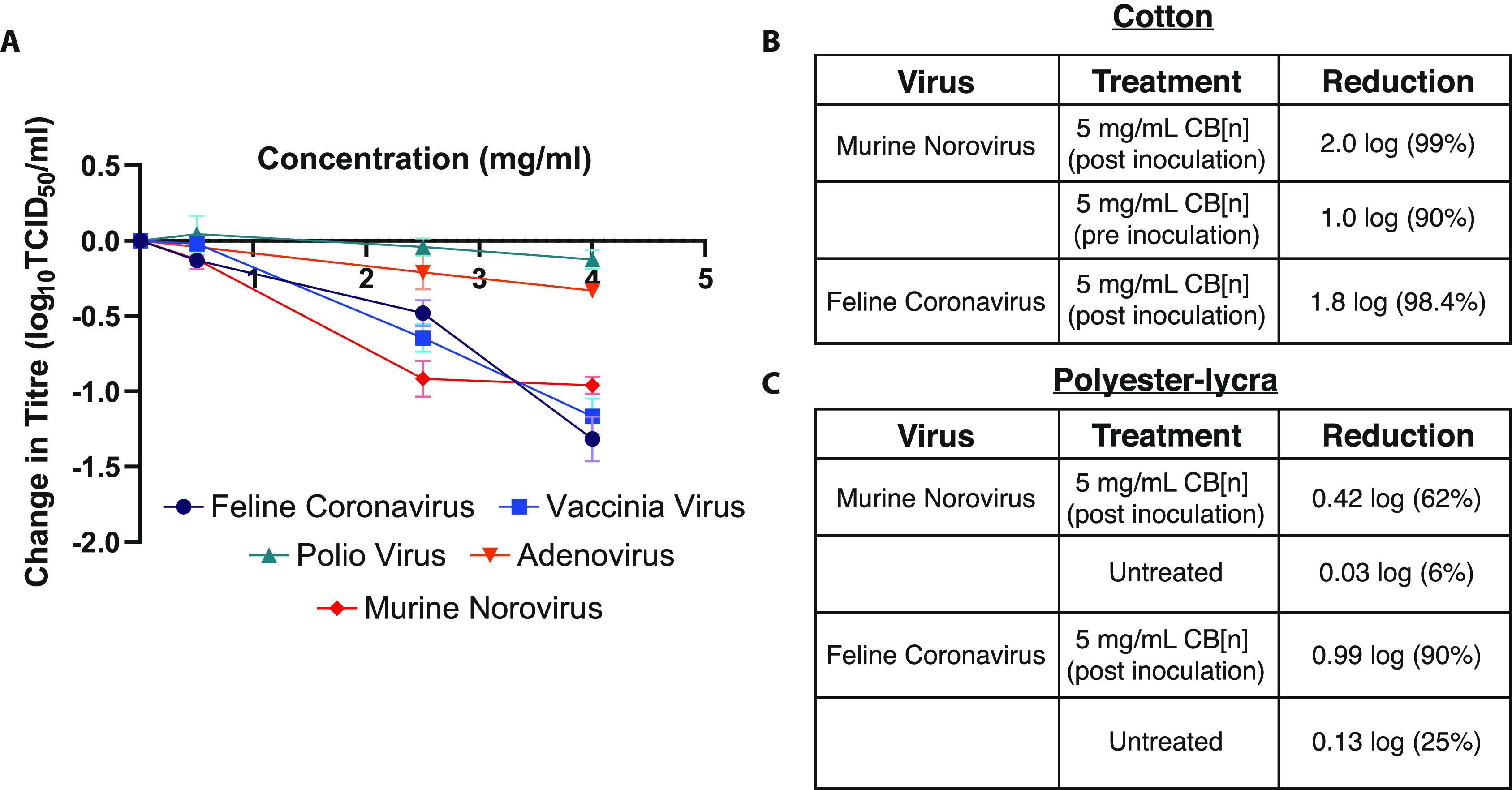
(A) TCID_50_ assays conducted according to EN 14476 were performed after mixing viral suspensions with antiviral suspensions. Of all five viruses tested, only feline coronavirus, vaccinia virus, and murine norovirus were inhibited by the CB[*n*] formulation (*n* = 2). (B) Surface testing of CB[*n*] formulations demonstrated some efficacy against murine norovirus and feline coronavirus. (C) Effect of pretreated polyester–lycra on murine norovirus and feline coronavirus.

To broaden the range of viruses tested against and further confirm the virucidal mode of action, a European standardized method was performed (EN 14476) in an accredited laboratory. EN 14476 specifically investigates products in suspension for use in disinfection in a medical context. Five viruses were investigated utilizing this method; poliovirus type 1, murine norovirus, adenovirus type 5, modified vaccinia virus, and feline coronavirus. A maximum of 5 mg/mL was used in this study to emulate concentrations used in CB-containing commercial products. The results from these experiments indicate that polio virus and adenovirus were not susceptible to CB[*n*], whereas feline coronavirus, vaccinia virus, and murine norovirus were susceptible to CB[*n*] (all three showed an approximately 1-log reduction in titer at the highest concentrations tested) (Figure 8A). Polio virus, adenovirus, and murine norovirus are all nonenveloped viruses; the presence or absence of an envelope is an important characteristic of a virus species and may be linked with the efficacy of CB[*n*]. Here, we observe that, while murine norovirus was inhibited by CB[*n*], polio virus and adenovirus were not.

As we had confirmed that the CB mode of action was virucidal, similar to disinfectants such as bleach, this broadens the scope of potential applications. Unlike bleach and other virucides, CBs are not damaging to surfaces such as textiles and have been demonstrated to not be irritating to skin or eye.^62^ Therefore, to determine their possible function as a surface disinfectant, another standardized testing assay (ISO 18184) was used and performed at an accredited laboratory. Murine norovirus and feline coronavirus were again used as surrogates for human norovirus and human coronaviruses. Figure 8B shows that a formulation containing 5 mg/mL CB[*n*] leads to a 98.4% reduction in feline coronavirus when added onto cotton inoculated with the virus (post inoculation) and a 99% reduction in murine norovirus (again post inoculation). It was also observed, with murine norovirus, that a 90% reduction in viral titer was achieved when the textile was pretreated with the CB[*n*] formulation (Figure 8B). In addition, further surface testing experiments were performed with treated fabric (polyester–lycra) at an accredited laboratory. These ISO 18184 surface tests again found a reduction in viral titer when the fabric was pretreated with 5 mg/mL CB[*n*]; feline coronavirus was reduced by 90% and murine norovirus, by 62% (Figure 8C).

## Discussion

Through these results, we have shown that cucurbit[*n*]urils are supramolecular broad-spectrum virucidal antivirals. Using HSV-2 as a model system, we confirmed antiviral efficacy, dose effect, and a virucidal mechanism. Using guests with a strong binding affinity for the CB cavity, we were able to show that this effect is on account of direct cavity binding to the virus. In addition, the fact the assays were performed in a media containing many different solutes confirms that a complex environment does not inhibit the antiviral effect. We were then able to broaden the range of viruses investigated to include several other species, including those of significant human health concern, such as RSV and SARS-CoV-2, again confirming an antiviral effect. Using solution and surface (textile) European standardized testing methods and surrogate viruses for some of the most dangerous human viruses, we further showed the significant potential of CBs as destroy-on-contact (virucidal) antivirals. This extracellular mode of action broadens the scope and real world uses of CB[*n*]s as antivirals to potentially include topical treatments, prophylaxes, soft surface (textile) disinfection, and aerosolization to deactivate airborne viruses.

## Methods

### Experimental Design

The study aimed to investigate whether cucurbiturils functioned as effective antiviral compounds through the use of various cellular assays. Once efficacy was demonstrated, further experiments were conducted to establish the mechanism by which cucurbit[7]uril acts as an antiviral. This involved a range of cellular assays but also a noncellular assay (DNA exposure).

### Cucurbituril Stocks

Cucurbituril stocks (CB[6], CB[7], and CB[8], and CB[*n*]) were provided courtesy of Aqdot Ltd. in dry powdered form. The substance CB[*n*] contains CBs 6, 7, and 8 in a 4:2:1 ratio. They were mixed with sterile deionized water (dH_2_O) to form an initial 50 mg/mL stock concentration (0.05 g of powder/mL dH_2_O, a 50 mg/mL solution). These were then diluted appropriately in sterile dH_2_O to form additional 20 mg/mL, 10 mg/mL, 5 mg/mL, and 0.5 mg/mL stocks. At 50 mg/mL, most stocks contained some undissolved CB, which sedimented over time. These stocks were always vortexed before experimental use to resuspend all the CB. dH_2_O was sterilized via filtration with a disposable syringe filter (Merck Millipore Ltd., Cork, Ireland) before use.

### Viruses

Herpes simplex virus, serotype two (HSV-2), and respiratory syncytial virus (RSV) samples were originally isolated, verified, and kindly donated by the University of Manchester School of Medical Sciences (Dr. Carol Yates). Additional stocks were grown on Vero cells in-lab and stored at −80 °C.

Murine norovirus (strain 1) was purchased from ATCC before stocks were grown on RAW 264.7 cells in-lab.

Coronavirus (strain OC43) was originally isolated, verified, and kindly donated by University of Manchester School of Biological Sciences (Prof. Pamela Vallely and Prof. Paul Klapper). Additional stocks were grown on MK1-Lu cells in-lab and stored at −80 °C. SARS-CoV-2 (strain SARS-CoV2/Switzerland/GE9586/2020) was obtained from a clinical specimen in the University Hospital in Geneva using Vero-E6 cells and passaged twice before use in the experiments. For the experiments, virus was propagated in Vero C1008 (clone E6) cells. Virus was handled appropriately in CL-3 laboratories.

### Cell Culture

All cell culture was performed using aseptic techniques in a class II microbiological safety cabinet (MBSC-II). All tissue culture media were supplemented with 1% penicillin/streptomycin (P/S) (Merck Life Science UK Ltd., Dorset, United Kingdom) and 10% heat inactivated fetal calf serum (FCS) (Merck Life Science UK) unless stated otherwise.

Vero cells were kindly donated by the University of Manchester School of Medical Sciences (Dr. Carol Yates). They were maintained in Dulbecco’s Modified Eagle Medium (DMEM) modified with high glucose, l-glutamine, phenol red, and sodium pyruvate (Thermo Fisher Scientific, Loughborough, UK). Cells were cultivated at 37 °C with 5% CO_2_ in 75 cm^2^ flasks and passaged in a 1:6 ratio when confluent. Vero C1008 (clone E6) cells, used for SARS-CoV-2 studies, were grown in DMEM modified with high glucose and Glutamax.

RAW 264.7 cells (Merck Life Science) were maintained in DMEM modified with phenol red and l-glutamine. Cells were cultivated at 37 °C with 5% CO_2_ in 75 cm^2^ flasks and passaged via cell scraping when confluent.

Mv-1 Lu cells (mink lung epithelial cells) were kindly donated by University of Manchester School of Biological Sciences (Dr. Pamela Vallely) and were maintained in DMEM modified with high glucose, sodium pyruvate, phenol red, and l-glutamine. Cells were cultivated at 37 °C with 5% CO_2_ in 75 cm^2^ flasks and passaged in a 1:6 ratio when confluent.

### Titration by the TCID_50_ Assay

Samples were titrated in 96-well flat bottom plates (Thermo Fisher Scientific) in quadruplicate. The first four wells of the first column were filled with 180 μL of appropriate media (see Cell Culture), and all other wells were filled with 100 μL of media containing the appropriate cells at a 2 × 10^5^/mL concentration. After cells adhered to the plate, wells A1–D1 then had 20 μL of sample added, before the contents were mixed via pipetting; 100 μL of mixed sample was then transferred to the next four wells and mixed again. This serial dilution was repeated across the plate with the exception of wells A12–D12, which were left unexposed to sample as a negative control. Plates were then incubated for an appropriate length of time depending on the virus being investigated. Virus titer was determined by cytopathic effects observed in wells via light microscopy examination. Titer was calculated using the Spearman and Käber method.

### Guest–Host Chemistry Assay

CB[7] powder stock was mixed with adamantylamine (Merck Life Science) and, separately, ferrocene (Merck Life Science) in a 1:1 molar ratio and resuspended at 50 mg/mL in sterile dH_2_O. Samples from this solution were then diluted further to form stocks of 50, 20, 10, 5, and 0.5 mg/mL.

### Dose–Response Assays

Cells were seeded at 170 000/mL with 500 μL of cell and media mixture added to each well of a 24-well plate (Corning, NY, USA). They were incubated at 37 °C overnight or until cells were 90–100% confluent. Sterile Eppendorf tubes were then prepared containing 570 μL of antiviral material mixed with DMEM to provide the desired concentration of antiviral. A 1:100 dilution of virus stock was also prepared; 30 μL of this diluted virus stock was added to each Eppendorf containing 570 μL of antiviral/media mix. An additional Eppendorf tube was also prepared containing just 570 μL of DMEM plus 30 μL of diluted virus stock to act as the nontreatment control (NTC). Eppendorfs containing antiviral–virus mixes and the NTC were incubated for 1 h at 37 °C. Then, all media were removed from the 24-well plate, and 200 μL of each antiviral–virus mix was added (in duplicate). The plate was then incubated for 1 h at 37 °C. Finally, the antiviral–virus mixes were removed by pipetting, and 500 μL of methylcellulose DMEM was added to each well. The plate was then incubated until visible plaques formed.

### Virucidal Assays

Cells appropriate to the virus were seeded into a 96-well plate at 15 000 cells per well and left until 90–100% confluent. Virus stock (55 μL) was incubated with the desired amount of antiviral agent to achieve the IC_90_ concentration in a 55 μL total volume (made up with phosphate buffered saline (PBS; Cambridge Bioscience, United Kingdom)). A nontreatment control was also created with 55 μL of virus stock mixed with 55 μL of PBS. Both virus plus antiviral and nontreatment controls were incubated for 1 h at 37 °C. Then, six 2 mL Eppendorf tubes were prepared, each containing 450 μL of the appropriate cell culture medium (three were used with the antiviral; three with the nontreatment control). After incubation, 50 μL was taken from the virus plus antiviral mix and added into the first Eppendorf tube before being resuspended 4–5 times. 50 μL was then serially diluted into the other two tubes, leaving three Eppendorfs at a 1:20, 1:200, and 1:2000 dilution, respectively. This process was also repeated for the nontreatment control. 50 μL from the virus plus antiviral mix (1:20 dilution) was then added to wells A1 and A2 of the 96-well plate. 50 μL from the 1:200 virus–antiviral mix was added to wells A3 and A4, and 50 μL from the 1:2000 virus–antiviral mix was added to wells A5 and A6. 50 μL from these wells was then serially diluted down the plate to row G. In well H1, 50 μL of the originally incubated virus plus antiviral stock was added, resuspended, and serially diluted to well H6. This process was repeated for the nontreatment control in wells A7–A12. The plate was then incubated for 1 h at 37 °C. All medium was then discarded and replated with 100 μL of methylcellulose DMEM. The plate was then incubated at 37 °C until visible plaques were formed; then, the plaques were stained with crystal violet to allow counting.

### Molecular Dynamic Simulations

MD simulations were performed with Nanoscale Molecular Dynamics version 2 (NAMD2). The CHARMM36 force field was used for the gB protein while the force field for the CB rings was taken from Gao et. al.^63^ The particle mesh Ewald (PME)^64^ method was applied for the assessment of long-range Coulombic interactions with a grid space of 1 Å. All simulations used the NPT ensemble, *p* = 1 atm, *T* = 310 K, γLang = 0.01 ps^–1^, and a time step of 2 fs. The systems were first minimized, heated, and then pre-equilibrated. All simulations were done for 100 ns, allowing the water and ions to settle (pre-equilibration) inside the rings and on the surface of the protein. MMGB-SA and NAMD energy were calculated in the last 20 ns of the simulations. The MM-GBSA free energy of binding between CB rings and gB proteins was evaluated by NAMD in a generalized Born Implicit Solvent (150 mm). The averaged MM-GBSA free energy was taken from 500 frames of each simulation as well as from NAMD energy. The trajectories and snapshots were visualized by VMD.^65^

### Genome Exposure Assay

#### Enzymatic Treatment

For HSV-2, 100 μL of viral sample was combined with 100 μL of antiviral sample in a sterile Eppendorf tube. A positive control was also included where 100 μL of virus stock is mixed with 100 μL of sterile deionized water. All samples were then incubated for 5 min at 37 °C in 5% CO_2_. Following co-incubation of virus and antiviral, DNA concentrations of each sample were measured using a NanoDrop Lite Spectrophotomer (ThermoFisher Scientific). The DNA or RNA concentration of each sample was noted. For DNA-genome viruses, 20 μL of 10× TURBO Dnase Buffer was added to each sample. In the sample with the highest amount of DNA (as measured by the spectrophotometer), 1 μL of TURBO Dnase was added per 1 μg of DNA present. An equal volume of TURBO Dnase was also added to all other samples in the batch. Samples were then incubated for 30 min at 37 °C in 5% CO_2_. Finally, EDTA (Merck Life Sciences) was added to each sample at a final concentration of 15 mM, and the samples were heated at 75 °C for 10 min to deactivate the TURBO Dnase.

#### Genome Extraction

Extraction was performed using a PureLink Viral RNA/DNA Mini Kit (Invitrogen) according to manufacturer’s instruction. Briefly, 25 μL of Proteinase K was added, before adding 200 μL of Viral Lysis Buffer. Samples were briefly vortexed and incubated at 56 °C for 15 min. 250 μL of molecular grade ethanol (Merck Life Sciences) was then added to each sample before a brief vortexing and incubation for 5 min at room temperature. Samples were then added to a Viral Spin Column in a collection tube before being centrifuged in a benchtop microcentrifuge at 6800*g* for 1 min (all subsequent centrifugation steps assume this speed unless otherwise specified). The flow-through was discarded, and 500 μL of wash buffer (WII) was added, before being centrifuged for 1 min. The flow-through was discarded again, and another 500 μL of wash buffer was added to each sample, before being centrifuged for 1 min. The spin column was then centrifuged in a clean collection tube for 1 min to remove any residual wash buffer (WII). Each spin column was then placed in a collection tube, and genetic material was eluted with 50 μL of sterile Rnase-free water. Samples were then incubated at room temperature for 1 min before being centrifuged for 1 min to elute nucleic acids. Purified viral DNA was stored at −80 °C before being amplified in qPCR.

### qPCR Amplification

qPCR was performed with a PowerUp SYBR Green Master Mix (Applied BioSystems). See Table 1 for primers used for qPCR; all primers used were purchased from Eurofins Europe. Samples for qPCR were made up to 20 μL total before being analyzed with the qPCR machine (Applied Biosystems StepOne Plus, ThermoFisher Scientific) and associated software. The PCR sequence used was as follows: holding stage (95 °C) for 10 min, followed by 40 cycles of 95 °C for 15 s and 60 °C for 1 min. This was followed by a single melt-curve stage of 95 °C for 15 s and 60 °C for 1 min.

**Table 1:** List of Primers used in the qPCR Experiment

virus	forward	reverse
HSV-2	GACAGCGAATTCGAGATGCTG	ATGTTGTACCCGGTCACGAACT

### Bacterial qPCR samples

Experiments were conducted using *Pseudomonas aeruginosa* (AP) strain ATCC PA01. *P. aeruginosa* was incubated overnight on nutrient agar plates. Bacterial DNA was extracted using a QIAamp DNA mini kit (Qiagen) using one bacterial colony. Primers for amplification were as follows: forward primer, 27F; reverse primer, 1492R (Eurofins Genomics).

### Time of Addition Study

Materials (10 or 20 μg) were added on cells 1 h before infection, during infection, or after infection with viruses added using a multiplicity of infection (MOI) of 0.01 using a method described previously.^22,66^ Viral titers were then quantified by the TCID_50_ assay.

### Immunofluorescence Experiment

Glass coverslips (13 mm diameter; Fisher Scientific) were added to 24-well plates (Corning Costar, Fisher Scientific). Cells of interest were seeded at a density of 2 × 10^5^/mL and left to adhere overnight at 37 °C with 5% CO_2_. A virus stock of known titer (approximately 6.0 log_10_TCID_50_/mL, added at an MOI of 0.02) was mixed with antiviral samples and added to cells for 60 min to allow for adsorption. After this, infectious medium was removed, and the well was washed with 1 mL of PBS applied by pipet before 1 mL of fresh tissue culture medium was added. After the required incubation period (24 h for HSV-2), the tissue culture medium was removed and the infected cells were fixed in 10% formalin for 1 h. After fixation, coverslips were transferred to a new 24-well plate and stored in PBS at 4 °C until labeling was performed. Labeling began with the removal of PBS and addition of 0.1% Triton X-100 (Merck Life Sciences) for approximately 15 min. Then, coverslips were washed once with PBS before nonspecific binding was blocked by the addition of 0.5% PBS/bovine serum albumin (BSA) for 30 min. 0.5% PBS/BSA was then removed, and primary antibody (anti-HSV-2 antibody 2C10, Abcam, United Kingdom), diluted in 0.5% PBS/BSA, was added for 60 min. Then, coverslips were washed three times in PBS before incubation with secondary antibody (Alexa Fluor 488, ThermoFisher Scientific) (diluted in 0.5% PBS/BSA) for 60 min. Next, coverslips were again washed three times in PBS, before being incubated with Alexa Fluor 568 Phalloidin (1:60 dilution) for 20 min. Phalloidin was then removed, and coverslips were incubated for 10 min in a 1:10 000 DAPI (Merck Life Sciences) solution (made up in deionized water). A drop of VECTASHIELD (Vectorlabs) mounting medium was then applied to a glass slide; coverslips were dipped in distilled water with fine forceps and then dried by being placed vertically on a paper towel. The coverslip was then placed, cells down, onto the mounting medium droplet. Coverslips were sealed by applying CoverGrip Sealant (Cambridge Bioscience) around the circumference, before being allowed to dry. Once dry, samples were stored in the dark at 4 °C before being viewed on a Leica confocal microscope with LAS X software (Leica Microsystems).

### Solution Testing (EN 14476)

A sample of the supplied test product (CB[*n*] commercial formulation Oderase) was diluted in distilled water. This was added to a test suspension of viruses in a solution of interfering substance (final concentration, 0.3 g/L bovine albumin). The mixture was maintained at 20 °C ± 1 °C and at different contact times (5 min and 1 h, data combined above). At the end of this contact time, an aliquot was taken and the virucidal action in this portion was immediately suppressed by a validated method (dilutions of the sample in ice-cold cell maintenance medium). The dilutions were transferred into cell-culture units using either a monolayer or cell suspension. Infectivity tests were done by either plaque test or quantal tests. After incubation, the titers of the infectivity were calculated according to Spearman and Käber or by plaque counting. The reduction of virus infectivity was calculated by the differences of log virus titers before (virus control) and after treatment with the supplied product. The spectrum of test organisms included poliovirus (Type 1KSc), adenovirus (Adenoid 75), murine norovirus (strain S99 Berlin), vaccinia virus (ATCC VR-5), and feline coronavirus (Munich strain).

### Textile Testing (ISO 18184)

A 20 mm × 20 mm sample of test material was cut (overall mass should be 0.40 g and was made up with extra layers of material as required). Nine control pieces were required, and 6 test pieces were used. Three pieces of each material were used to test the effect of the fabric on cells without virus (cytotoxicity), and 3 control pieces were used to recover the starting titer of the virus. The remaining pieces were inoculated with 200 μL of virus at a concentration of ∼10^7^ TCID_50_ (giving a final concentration of 10^5^) and left for the contact time of 2 h. Following the contact time, the fabric was recovered in 20 mL of cell culture media and enumerated onto an appropriate cell line. TCID_50_ was calculated following the appropriate incubation time. Antiviral activity was calculated by a comparison of the antiviral test material to the immediate recovery from the control fabric.

Additional textile testing was carried out on polyester–lycra samples. In these instances, impregnated CB[*n*] textile was prepared by immersing samples of polyester–lycra in a 5 wt % CB[*n*] suspension in water (1 L in 3 L beaker) for 10 min while mixing at 300 rpm using a magnetic stirrer. Wet textile samples were recovered and then squeezed between two rollers to remove excess solution until no further solution could be removed. Textile samples were then dried in a convection oven at 110 °C for 15 min. The CB[*n*] loading of the dried textile was gravimetrically determined to be 5 wt % on average. The control textiles were immersed in water and treated by the same procedure. Antiviral tests were conducted following ISO 18184.

### Statistical Analysis

Statistical analysis (for the generation of IC_50_ values) was performed using GraphPad Prism software, version 9.1.1, and a nonlinear fit of [inhibitor] vs response–variable slope (four parameters). Furthermore, different cucurbituril treatments in the TCID_50_ assays were analyzed for statistical differences using the same version of GraphPad Prism software and a two-way ANOVA with Tukey’s multiple comparisons. A one-way ANOVA was used to compare the fold-change in genomic material detection in Figure 5B and viral titers in Figure 5C.

## Supporting Information Available

S1, confocal immunofluorescence microscopy images of Vero cell monolayers after 24 h of exposure to CB[*n*] mixed with HSV-2; S2, qPCR controls to determine the effect of the CBs on Ct values; S3, virucidal assay against RSV; S4, TCID_50_ assay illustrating the effect of the CBs against MNV1; Appendix 1, additional confocal immunofluorescence microscopy images for each tested condition of CB[7] with HSV-2; Appendix 2, additional confocal immunofluorescence microscopy images for each tested condition of CB[*n*] with HSV-2 (PDF)

## Notes

All data needed to evaluate the conclusions in the paper are present in the paper and/or the Supporting Information. Additional data related to this paper may be requested from the authors.
