# Supplementary material for: Broad-Spectrum Extracellular Antiviral Properties of Cucurbit[n]urils
Source: ACS Infect Dis. 2022 Sep 5;8(10):2084–95. doi: 10.1021/acsinfecdis.2c00186 (PMC9578052; doi:10.1021/acsinfecdis.2c00186)
Supplement: Supplementary file 1 — id2c00186_si_001.pdf [file id2c00186_si_001.pdf]

Broad-spectrum extracellular antiviral properties of Cucurbit[*n*]urils.

Authors
Luke M. Jones[1], Elana H. Super[1], Lauren J. Batt[1], Matteo Gasbarri[2], Francesco Coppola[3], Lorraine M. Bhebhe[1], Benjamin T. Cheesman[4], Andrew M. Howe[4], Petr Kral[5], Roger Coulston[4], Samuel T. Jones[1]*

Affiliations
1. Department of Materials and The Henry Royce Institute, The University of Manchester, Manchester, M19 3PL, UK
2. Institute of Materials, Interfaculty Bioengineering Institute, MXG 030, Lausanne, Switzerland
3. Department of Chemistry, University of Illinois at Chicago, Chicago, IL, 60607, USA
4. Aqdot Limited, Iconix Park, London Road, Pampisford, Cambridge, CB22 3EG, UK
5. Department of Chemistry, Physics and Department of Biopharmaceutical Sciences, University of Illinois at Chicago, Chicago, IL, 60607, USA

*samuel.jones-4@manchester.ac.uk

Confocal Immunofluorescence Microscopy images of Vero cell monolayers after 24hr exposure to CB[*n*] mixed with HSV-2

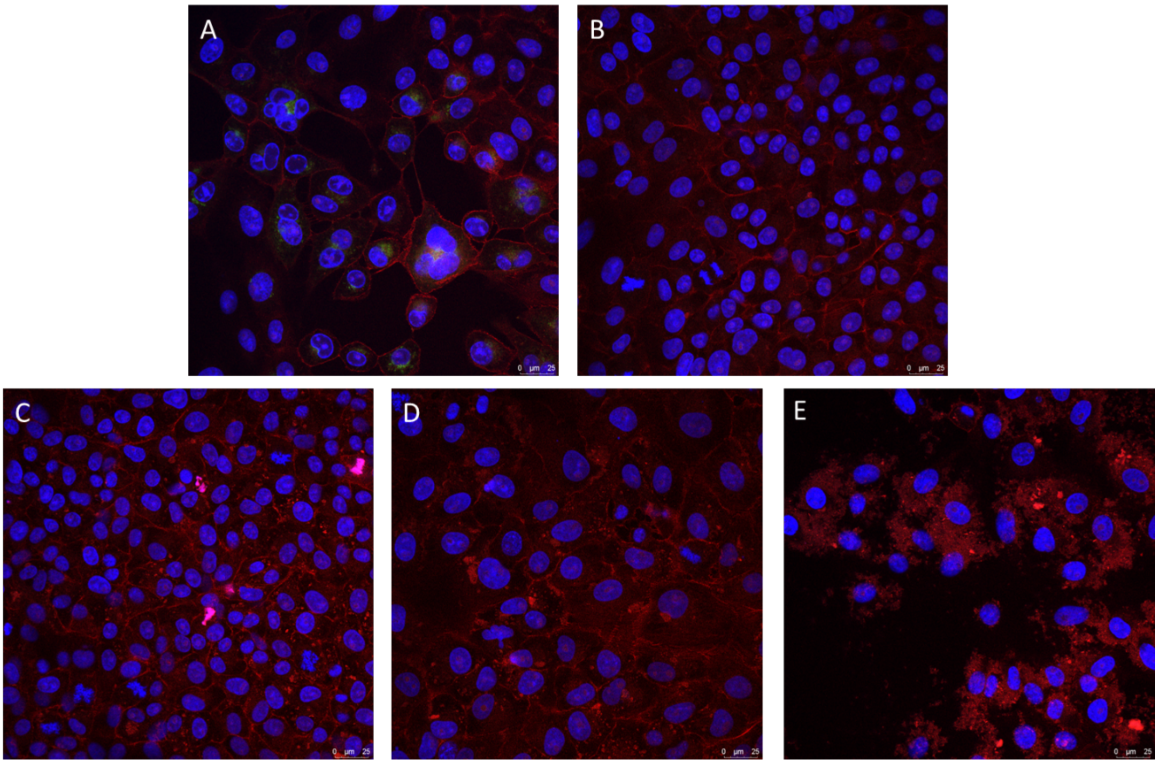

Figure S1: HSV-2 was mixed with different concentrations of CB[*n*] (A: 0 mg/mL, B: 5 mg/mL, C: 10 mg/mL, D: 20 mg/mL, E: 50 mg/mL) and applied to cells, before incubating for 24 hours. Visual indications are that CB[*n*] has an antiviral effect at 5 mg/mL concentrations and above. However, at 20 mg/mL and particularly 50 mg/mL, cellular damage becomes increasingly visible, with the monolayer broken apart at the highest concentrations (E). Cell nuclei in blue, phalloidin in red, HSV-2 in green.

Molecular Dynamic Simulations

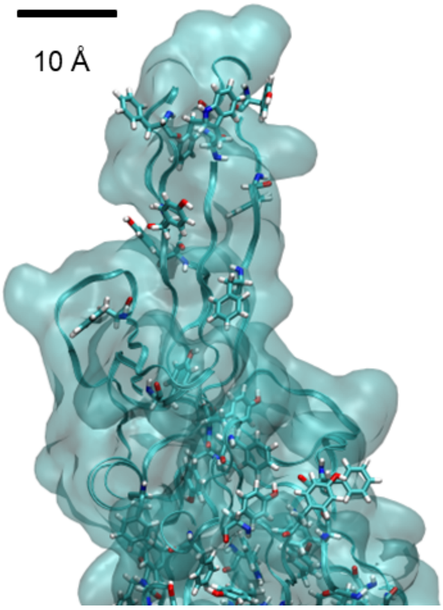

Figure S2: Simulation of the aromatic residues of the HSV-2 gB protein. Given their position and structure on the surface, they are not easily accessible.

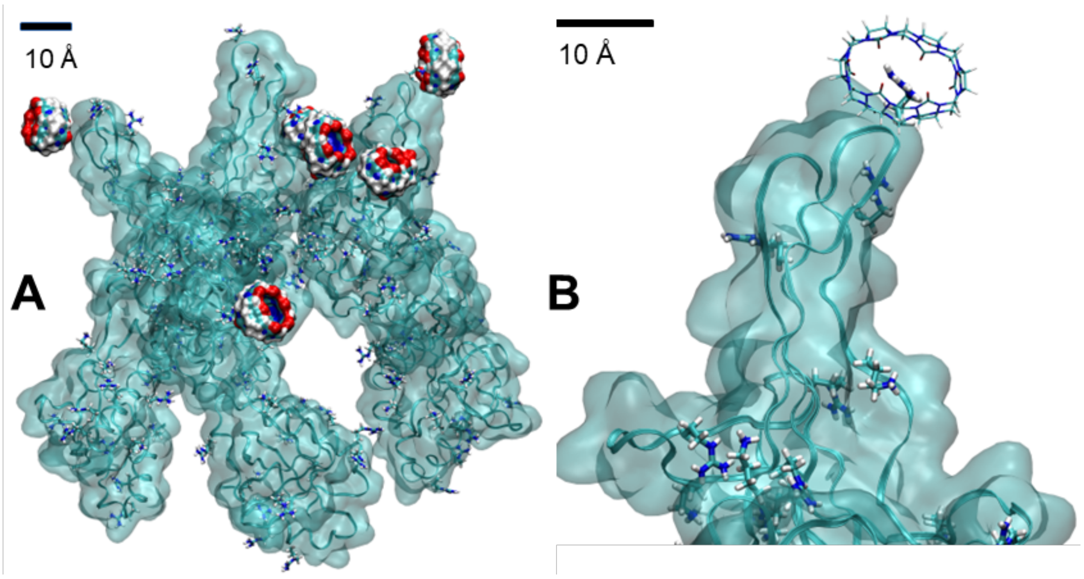

Figure S3: A) Shows the CB[6] interacting with the basic amino acids present on the gB protein of HSV-2. B) Detailed view of how CB[6] encapsulates ARG.

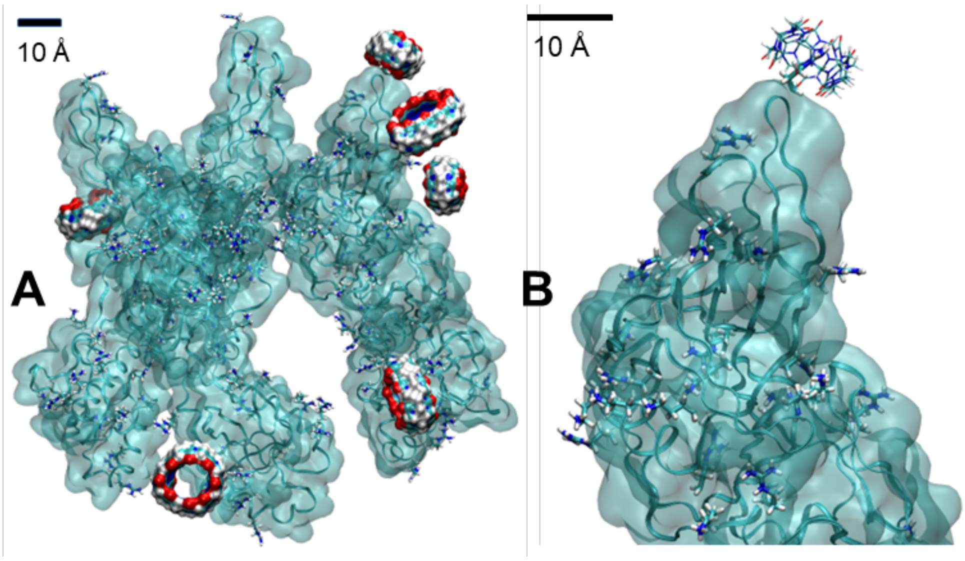

Figure S4: A) Shows the CB[8] interacting with the basic amino acids present on the gB protein of HSV-2. B) detailed view of how CB[8] encapsulates ARG.

<u>qPCR Controls to determine the effect of CBs on Ct values</u>

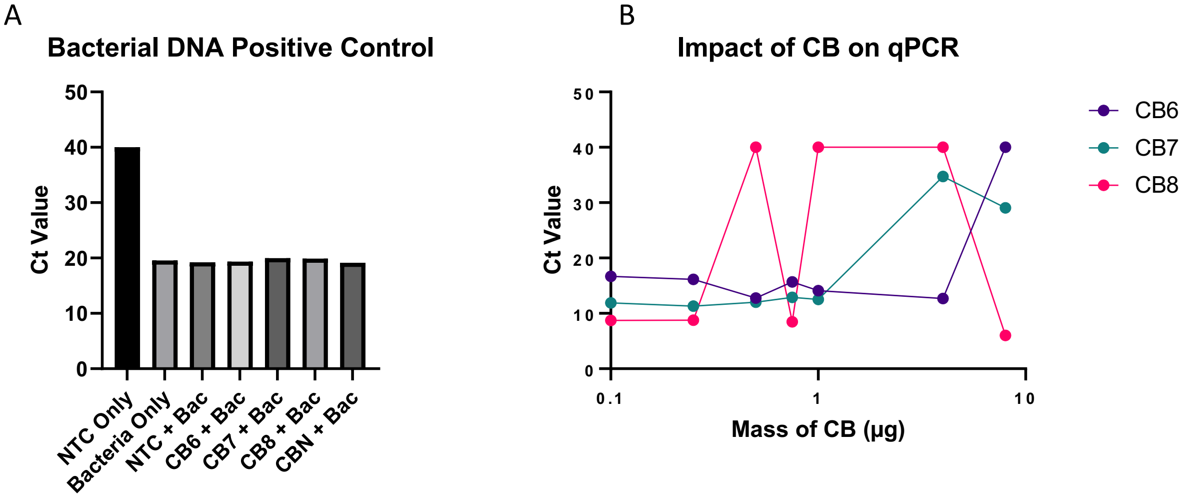

Figure S5: A) Bacterial DNA and primers were run as a positive control for qPCR. PCR primers did not amplify extracted HSV-2 viral DNA (left most bar). On all other occasions, extracted viral DNA samples, that had initially been mixed 1:1 with cucurbiturils, did not contain enough cucurbituril to artificially alter the Ct value. B) Interestingly, when cucurbiturils are added directly to the PCR mastermix, they can alter the Ct value, though only above certain concentrations (which vary depending on the homologue). For this experiment CBs were added directly to PCR master mixes containing extracted HSV-2 DNA (that had not been exposed to any cucurbituril treatment).

Virucidal RSV data

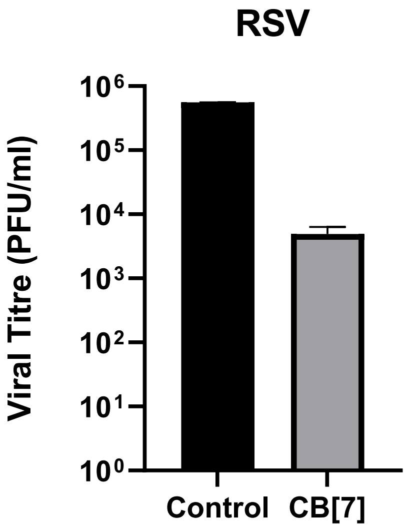

Figure S6: Virucidal assay performed against respiratory syncytial virus (RSV). A virucidal assay is used to distinguish between destructive (virucidal) and non-destructive (virustatic) interactions between antivirals and viruses. In this instance a two log-reduction was observed in RSV titre when 7.5 mg/mL of CB[7] was utilised (approximately IC90), indicating a virucidal mode of action.

TCID$_{50}$ Assay illustrating effect of CBs on MNV1

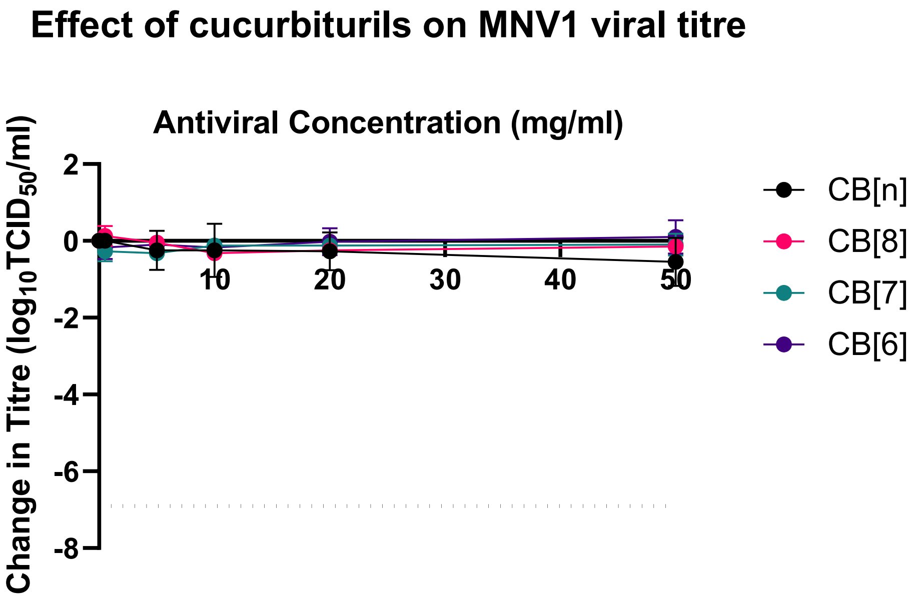

Figure S7: No cucurbituril homologue appeared effective against MNV1 in TCID$_{50}$ assays (n=3 for all homologues).

Additional Confocal Immunofluorescence Microscopy images for each tested condition of CB[7] with HSV-2

NTC – Scale Bars = 25 µm

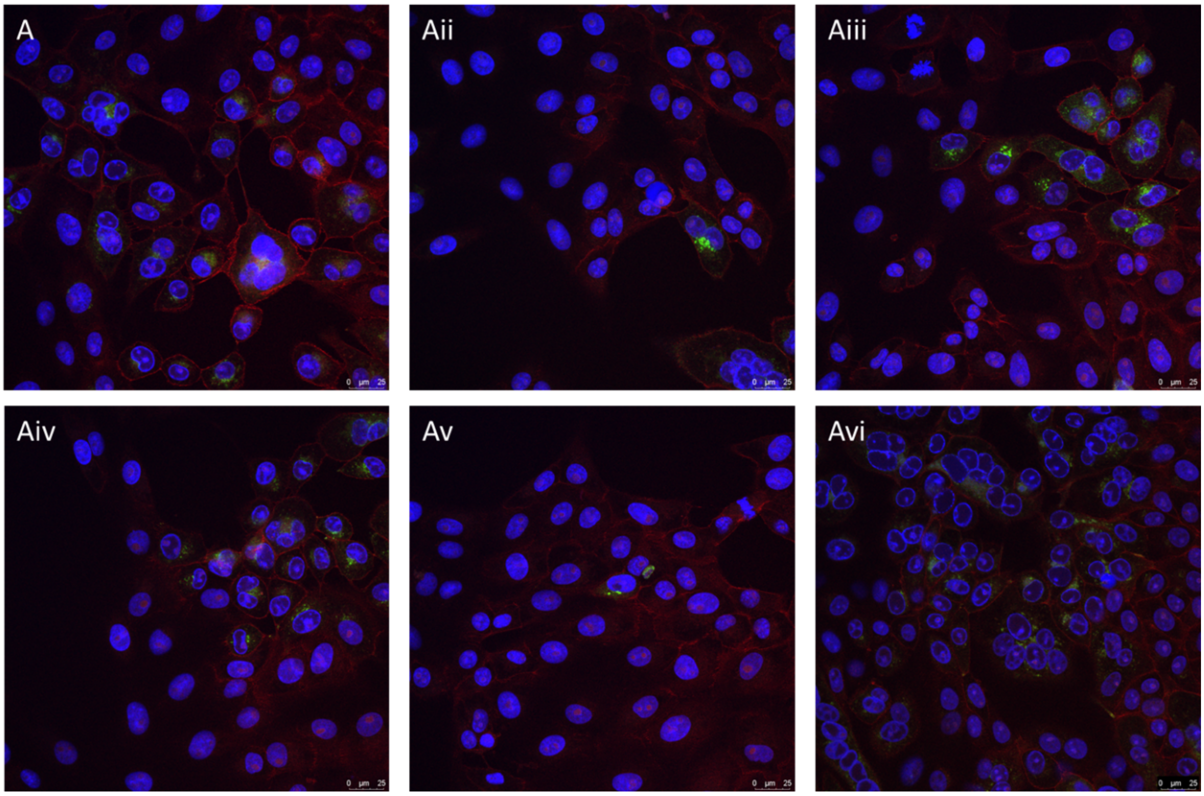

0.05%wt (0.5 mg/mlL) CB[7] – Scale Bars = 25µm

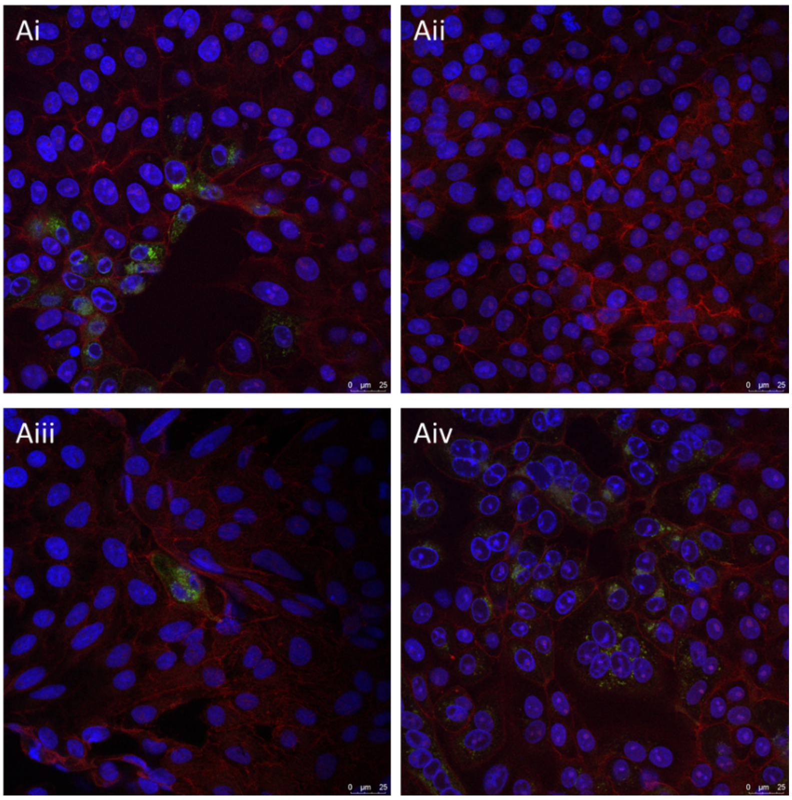

0.5wt% (5 mg/mL) CB[*n*] – Scale Bars = 25 µm

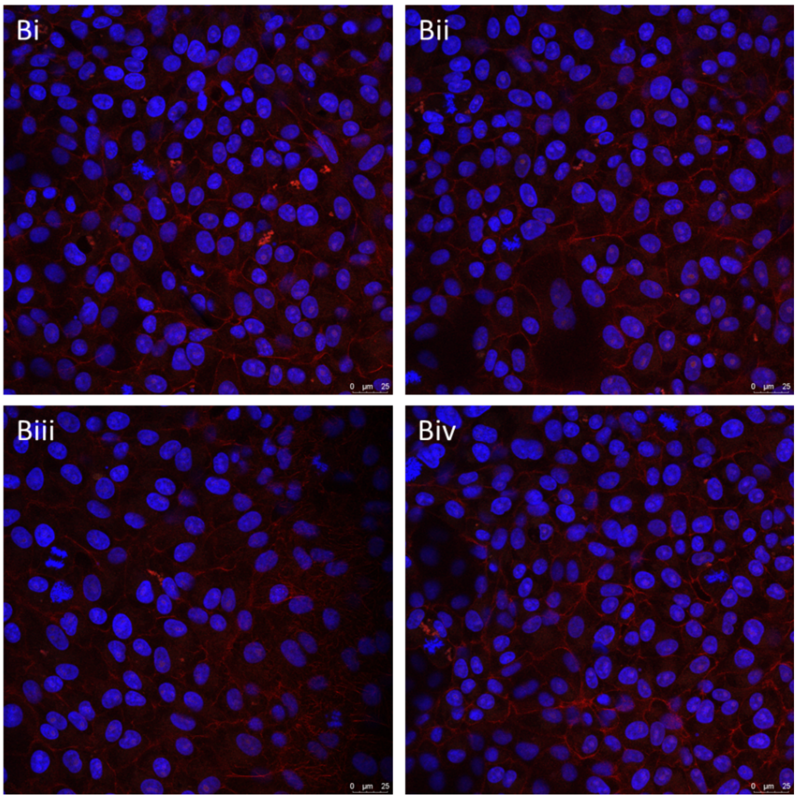

1wt% (10 mg/mL) CB[*n*] – Scale Bars = 25 µm

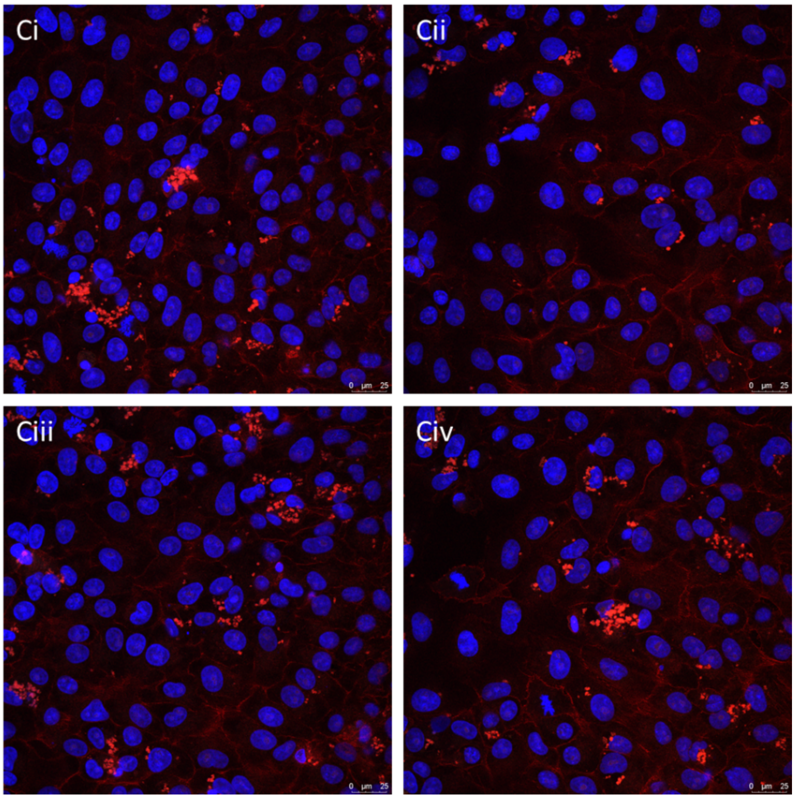

2wt% (20 mg/mL) CB[*n*] – Scale Bars = 25 µm

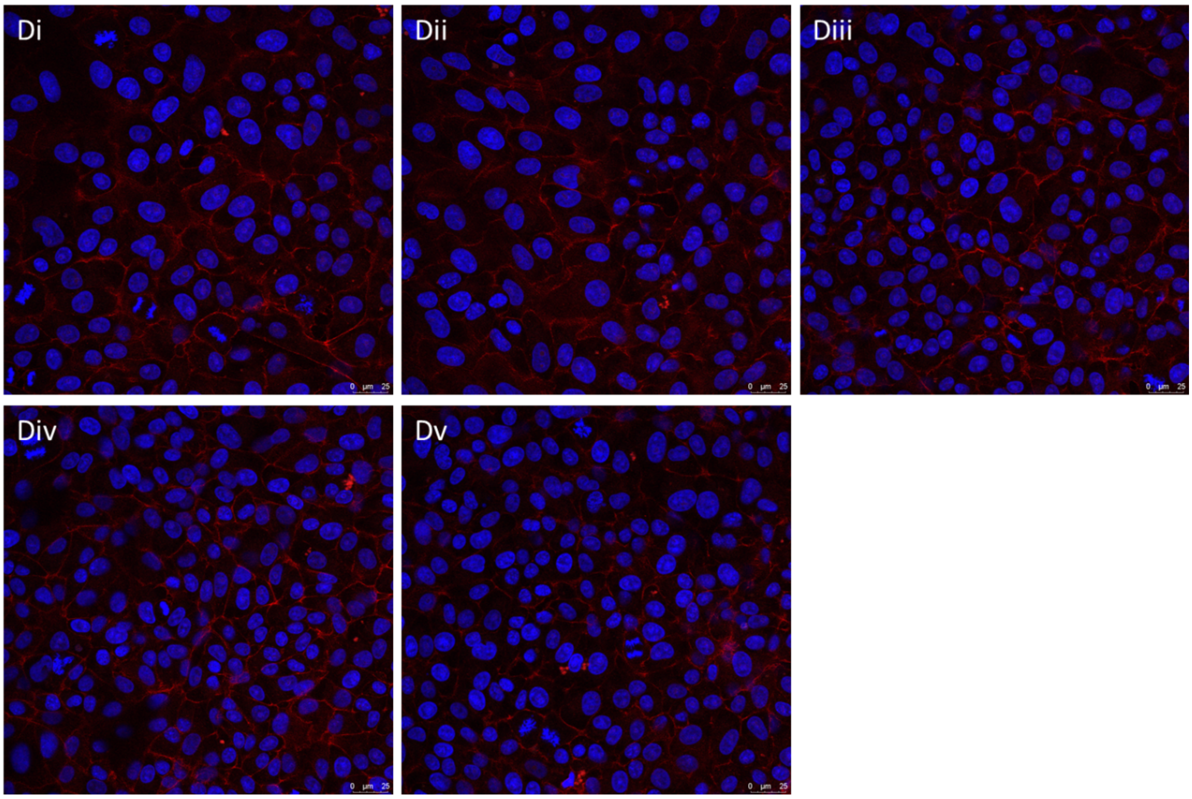

<u>5wt% (50 mg/mL) CB[*n*] – Scale Bars = 25 µm</u>

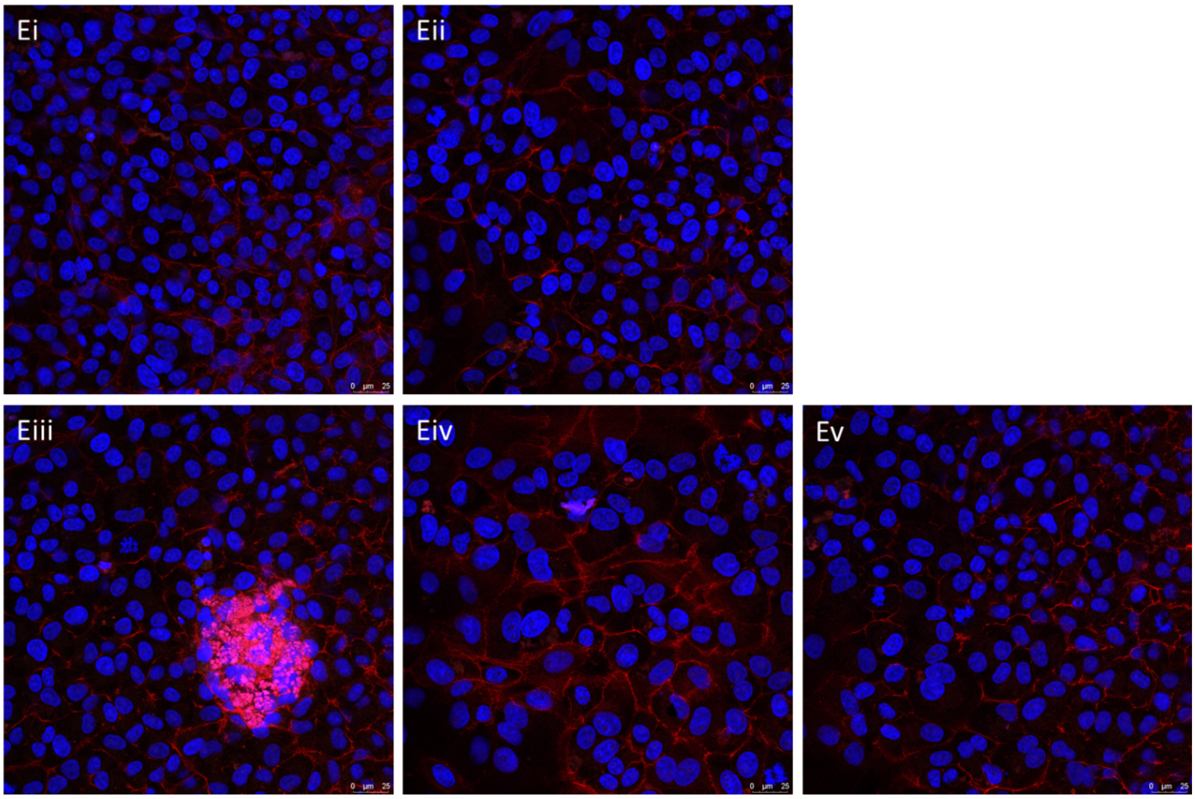

Appendix 1: Additional images of each tested condition of CB[7] with HSV-2. A: No-treatment control. B, 5 mg/mL CB[7]. C, 10 mg/mL CB[7]. D, 20 mg/mL CB[7]. E, 50 mg/mL CB[7].

<u>Additional Confocal Immunofluorescence Microscopy images for each tested condition of CB[*n*] with HSV-2</u>

<u>NTC – Scale Bars = 25 μm</u>

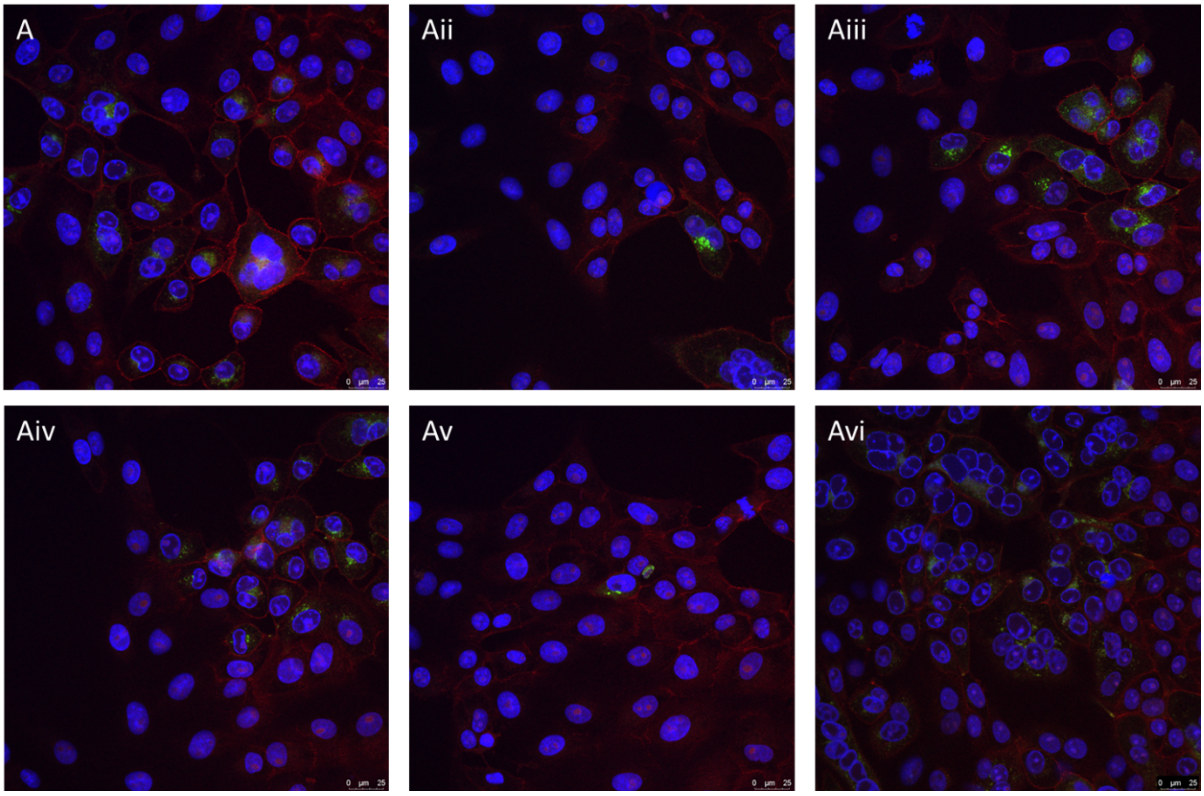

0.5wt% (5 mg/mL) CB[*n*] – Scale Bars = 25 µm

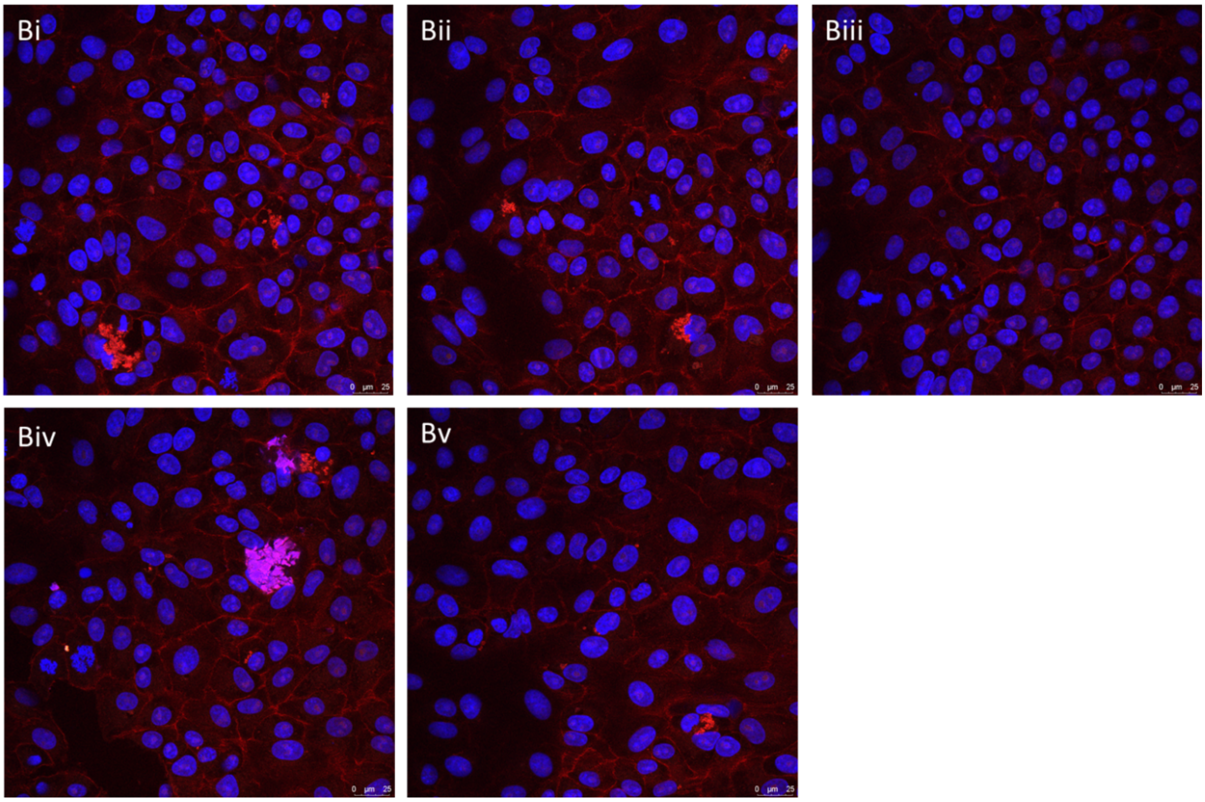

1wt% (10 mg/mL) CB[*n*] – Scale Bars = 25 µm

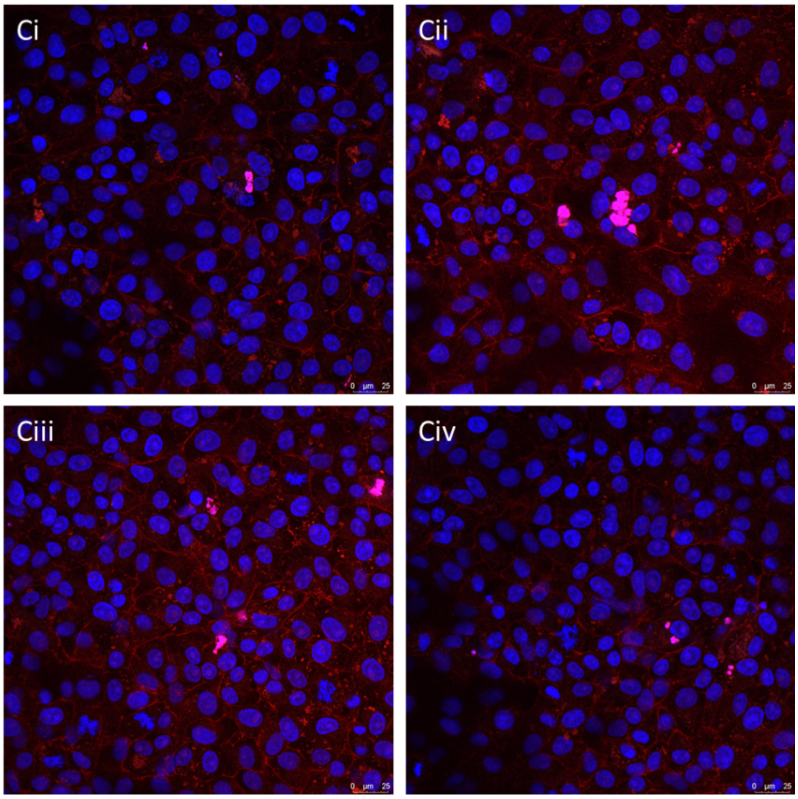

2wt% (20 mg/mL) CB[*n*] – Scale Bars = 25 μm

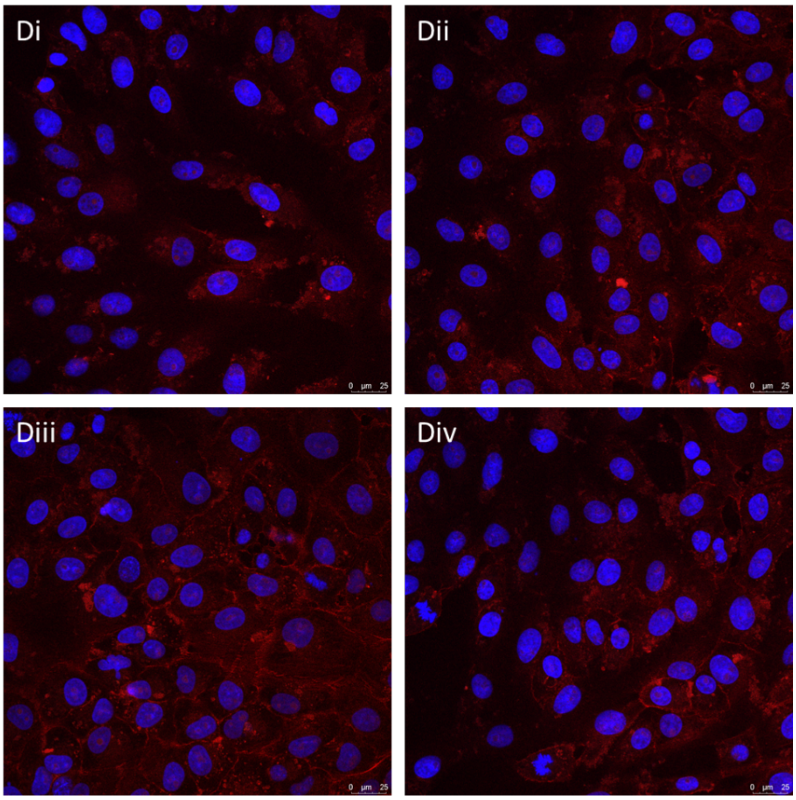

5wt% (50 mg/mL) CB[*n*] – Scale Bars = 25 μm

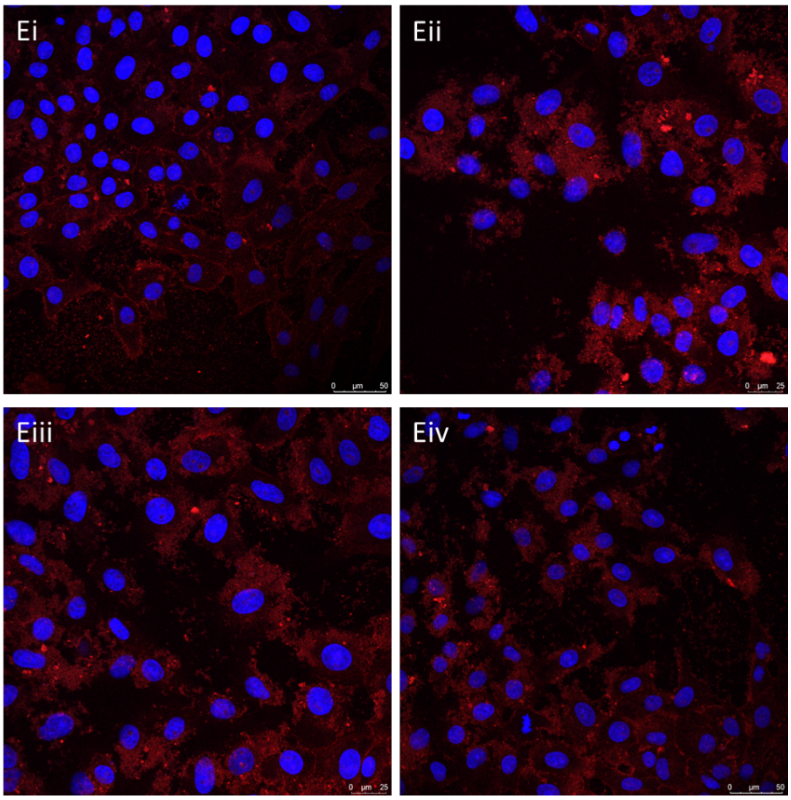

Appendix 2: Additional images of each tested condition of CB[n] with HSV-2. A: No-treatment control. B, 5 mg/mL CB[*n*]. C, 10 mg/mL CB[*n*]. D, 20 mg/mL CB[*n*]. E, 50 mg/mL CB[*n*].
